# Olfactory Proteins and Their Expression Profiles in the *Eucalyptus* Pest *Endoclita signifier* Larvae

**DOI:** 10.3389/fphys.2021.682537

**Published:** 2021-07-19

**Authors:** Xiaoyu Zhang, Zhende Yang, Xiuhao Yang, Hongxuan Ma, Xiumei Liu, Ping Hu

**Affiliations:** ^1^Forestry College, Guangxi University, Nanning, China; ^2^Forestry College, Central South University of Forestry and Technology, Changsha, China; ^3^Guangxi Academy of Forestry, Nanning, China; ^4^GuangxiGaofeng National Forest Farm, Nanning, China

**Keywords:** expression profiles, chemosensory proteins, transcriptome, odorant binding protein, *Endoclita signifier*

## Abstract

*Endoclita signifier* Walker (Lepidoptera: Hepialidae), a polyphagous insect, has become a new wood-boring pest in Eucalyptus plantations in southern China since 2007, which represents a typical example of native insect adaptation to an exotic host. After the third instar, larvae move from soil to standing trees and damage the plants with a wormhole. Although females disperse to lay eggs, larvae can accurately find eucalyptus in a mingled forest of eight species, which leads us to hypothesize that the larval olfactory system contributes to its host selection. Herein, we investigated the transcriptomes of the head and tegument of *E. signifer* larvae and explored the expression profiles of olfactory proteins. We identified 15 odorant-binding proteins (OBPs), including seven general OBPs (GOPBs), six chemosensory proteins (CSPs), two odorant receptors (ORs), one gustatory receptor (GR), 14 ionotropic receptors (IRs), and one sensory neuron membrane protein (SNMP). Expression profiles indicated that all olfactory proteins, except for EsigCSP1, were expressed in the head, and most were also detected in non-olfactory tissues, especially thorax tegument. Furthermore, EsigOBP2, EsigOBP8, EsigGOBP1, EsigGOBP2, EsigGOBP5, EsigCSP3, EsigCSP5, and EsigOR1 were expressed most strongly in the head; moreover, EsigCSP3 expressed abundantly in the head. EsigGR1 exhibited the highest expression among all tissues. Besides phylogenetic analysis shows that EsigGOBP7 probably is the pheromone-binding protein (PBP) of *E. signifier*. This study provides the molecular basis for future study of chemosensation in *E. signifier* larvae. EsigCSP3 and EsigGR1, which have unique expression patterns, might be factors that govern the host choice of larvae and worth further exploration.

## Introduction

**Figure 1:**
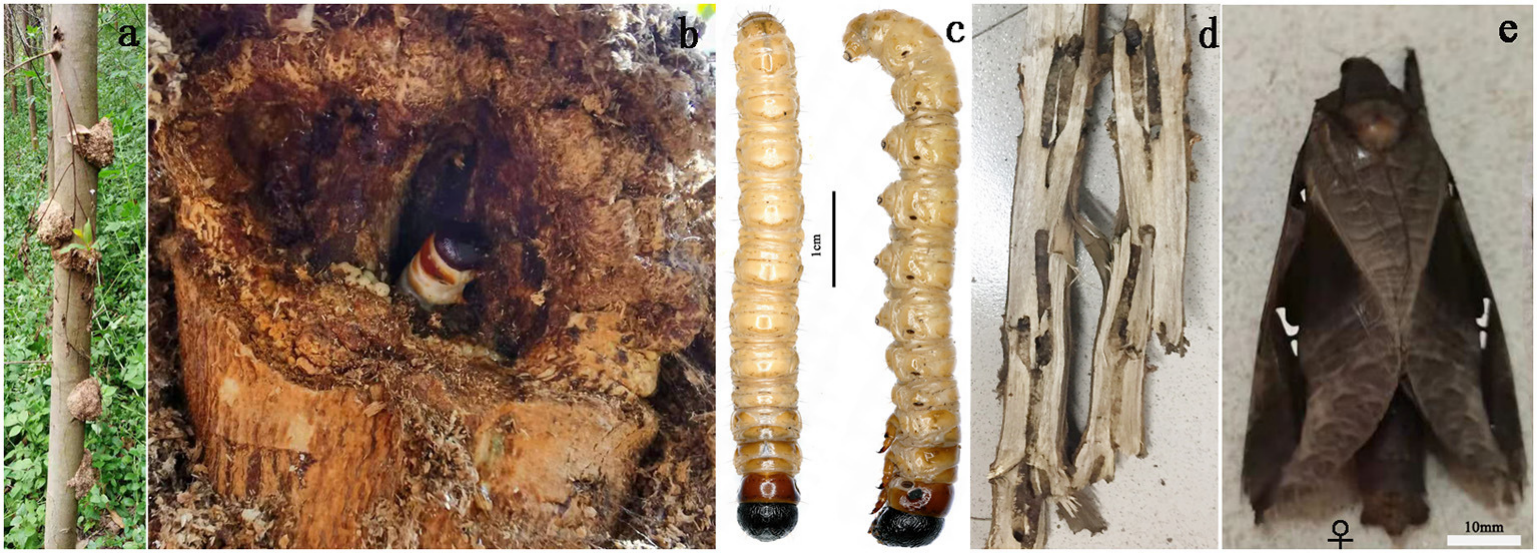
**(a,b,d)** Damage caused by *E. signifer*. **(c)** Larvae and **(e)** adults of *E. signifer*.

The ghost moth *Endoclita signifer* Walker (Lepidoptera, Hepialidae) is a native polyphagous insect pest that is widely distributed in Japan, Korea, India, Thailand, Myanmar, and central, south, and southwest China (Yang et al., 2021). After *Eucalyptus* was planted in the south of China, *E*. *signifer* was discovered to have infested *Eucalyptus* in Guangxi in 2007. This is an example of a native insect adapting to an exotic host, and it has resulted in great economic losses and major ecological impacts (Yang et al., 2016). In 2020, an infestation of *E. signifier* was found in 17.1% of the counties in Guangxi, where host plants included 51 species in 40 genera and 30 families (Yang et al., 2021). In Guangxi, *E*. *signifer* usually produces one generation a year and occasionally two. The adults eclose from mid-March through April and then mate and oviposit. Larvae hatch within 1 month and live in the soil. After the third instar stage, from July through August, the larvae move from soil to standing trees, where they feed on bark, bore into the stem, and weave wood pieces and silk over the hole entrance, constructing a home in which they reside until the following January before pupating in February (Figure 1). Some larvae spend two years in the tree, until January of the third year (Yang, 2013). Although female oviposition proceeds in a dispersed manner, larvae were shown to damage eight *Eucalyptus* species in mixed forests (Yang, 2017). Besides, the sensillum of *E. signifier* larva shows that only 16 sensilla in antennae, but a large number of sensilla in thoracic and abdominal tegument (Hu et al., 2021). Therefore, we hypothesized that the thoracic and abdominal tegument may have an olfactory function and help larvae to find their host.

In most insects, especially stem borers, females select the host. Olfaction plays a minor role in larvae. The small number of neurons in the *Drosophila* olfactory system makes it a very convenient system for olfactory studies (Bose et al., 2015), enabling the exploration of sensory coding at all stages of nervous system development (Bose et al., 2015). In *Drosophila* larvae, the epidermal growth factor receptor (Rahn et al., 2013) and serotonin signaling (Annina et al., 2017) are necessary for learning and memory. However, sub-circuits allow *Drosophila* larvae to integrate present sensory input into the context of past experience and elicit an appropriate behavioral response (Rahn et al., 2013). The responsiveness of larval sensilla to female-emitted sex pheromones is based on the same molecular machinery as that functioning in the antennae of adult males (Zielonka et al., 2016). Olfactory proteins that bind, transport, and degrade odor molecules play important roles in chemo-sensing in larvae and include odorant-binding proteins (OBPs), chemosensory proteins (CSPs), sensory neuron membrane proteins (SNMPs), odorant receptors (ORs), gustatory receptors (GRs), ionotropic receptors (IRs), and odor-degrading enzymes (ODEs) (Zhou, 2010). Behavior, physiology, and molecular result indicated olfactory proteins of larvae play a role in pheromone or volatile recognition. For example, four CSPs in *Chilo auricilius* larvae (Yi et al., 2019) were significantly regulated by α-pinene treatment. Pre-exposure larvae to single volatile organic compounds can upregulate ORs in *Spodoptera exigua* larvae strongly and non-specifically (Llopis-Gimenez et al., 2020). *S. exigua* larval OBP can bind to a major female sex pheromone component (Jin et al., 2015), whereas OBP8 of *Melipona scutellaris* is expressed and functioned in the larval mandible (Carvalho et al., 2017).

This study investigated the transcriptomes in the head and tegument of *E*. *signifer* larvae to determine the expression profiles of olfactory proteins in *E*. *signifer* larvae and evaluated the phylogenetic relationships between *E*. *signifer* OBPs and CSPs with those expressed in the larvae and adults of other species. This work illuminates the olfactory system in *E*. *signifer* larvae and provides a theoretical basis for further studies to explore ecologically relevant larval behaviors.

## Materials and Methods

### Ethics Statement

The ghost moth *E. signifier* is a forestry pest in China, which were collected with the direct permission of the Guangxi forestry bureau. It is not included in the “list of endangered and protected animals in China.” All operations were performed according to ethical guidelines in order to minimize pain and discomfort to the insects.

### Insect and Tissue Collection

*E. signifier* larvae were collected from damaging *Eucalyptus* plantation by cutting the tree during December 2019 to January 2020 and September to November 2020 in the Gaofeng forest station, Guangxi, China. Six 12th larvae and 18 5th larvae were taken indoors and stored at −80°C. Larval thoracic and abdominal tegument were obtained by using surgical scissors to cut open the larvae abdomen through the midline and using tweezers to remove from the rest of the body (intestinal canal and fat body), and the teguments were cleaned in RNA-free ddH_2_O.

### cDNA Library Construction and Illumina Sequencing

Total RNA was extracted from six 12th larval head, thoracic, and abdominal tegument using TRIzol reagent (Ambion) and the RNeasy Plus Mini Kit (No. 74134; Qiagen, Hilden, Germany), following the instructions of the manufacturer, respectively. RNA quantity was detected using the NanoDrop 8000 (Thermo, Waltham, MA, USA). There is one replication, each replication with six larvae. RNA of 12th larval heads and thoracic and abdominal tegument were used to construct the cDNA libraries. cDNA library construction and Illumina sequencing of samples were performed at MajorBioCorporation (Shanghai, China). mRNA samples were purified and fragmented using the TruSeq RNA Sample Preparation Kit v2-Set A (No. RS-122-2001; Illumina, San Diego, CA, USA). Random hexamer primers were used to synthesize the first-strand cDNA, followed by synthesis of the second-strand cDNA using a buffer, dNTPs, RNase H, and DNA polymerase I at 16°C for 1 h. After end repair, A-tailing, and the ligation of adaptors, the products were amplified by PCR and quantified precisely using the Qubit DNA Br Assay Kit (Q10211; *Invitrogen*, Carlsbad, CA, USA). They were then purified using the MinElute Gel Extraction Kit (Qiagen, Cat No. 28604) to obtain a cDNA library. The cDNA library was sequenced on the HiSeq2500 platform.

### Assembly and Functional Annotation

All raw reads were processed to remove low-quality and adaptor sequences by Trimmomatic (http://www.usadellab.org/cms/index.php?page=trimmomatic). Clean reads assembly was carried out with the short-read assembly program Trinity (Version: r2020-01-13), with the default parameters, after combining the heads and thoracic and abdominal tegument clean reads. The largest alternative splicing variants in the Trinity results were called “unigenes.” The annotation of unigenes was performed by NCBI BLASTx searches against the Nr protein database, with an E-value threshold of 1e-5. The blast results were then imported into the Blast2GO pipeline for GO annotation. The longest ORF for each unigene was determined by the NCBI ORF Finder tool (http://www.ncbi.nlm.nih.gov/gorf/gorf.html). Expression levels were expressed in terms of FPKM values (Mortazavi et al., 2008), which were calculated by RSEM (RNA-Seq by Expectation-Maximization) (Version: v1.2.6) with default parameters (Li and Dewey, 2011).

### Identification of Chemosensory Genes

With BLASTx, the available sequences of OBPs, CSPs, ORs, GRs, IRs, and SNMPs proteins from insect species were used as queries to identify candidate unigenes involved in olfaction in *E. signifier* from the Nr database. All candidate OBPs, CSPs, ORs, GRs, IRs, and SNMPs were manually checked by tBLASTn in NCBI online by assessing the BLASTx results. The nucleic acid sequences encoded by all chemosensory genes that were identified from the *E. signifier* larval head and thorax and abdomen tegument transcriptome are listed in Supplementary Material 1.

### Sequence and Phylogenetic Analysis

The candidate OBPs were searched for the presence of N-terminal signal peptides using SignalP4.0 (http://www.cbs.dtu.dk/services/SignalP/). Amino acid sequence alignment was performed using the muscle method implemented in the Mega v6.0 software package (Tamura et al., 2011). The phylogenetic tree was constructed using the neighbor-joining (NJ) method (Saitou and Nei, 1987) with the P-distances model and a pairwise deletion of gaps performed in the Mega v6.0 software package. The reliability of the tree structure and node support was evaluated by bootstrap analysis with 1,000 replicates. The phylogenetic trees were colored and arranged in FigTree (Version 1.4.2). The phylogenetic analyses of *OBPs* were based on *Dastarcus helophoroides* (Li et al., 2020), *Chrysomya megacephala* (Wang et al., 2015)*, Plutella xylostella* (Zhu et al., 2016), *S. exigua* (Liu et al., 2015; Llopis-Gimenez et al., 2020), *Helicoverpa armigera* (Chang et al., 2017), and *E. signifier*. The CSPs tree was based on *D. helophoroides* (Li et al., 2020), *C. megacephala* (Wang et al., 2015)*, S. exigua* (Llopis-Gimenez et al., 2020), *H. armigera* (Chang et al., 2017), and *E. signifier*. The gene name and the Genbank number of *P. xylostella* and *H. armigera* are listed in Supplementary Material 2; other genes sequences are available in the reference article.

### Expression Analysis by Fluorescence Quantitative Real-Time PCR

Fluorescence quantitative real-time PCR was performed to verify the expression of candidate chemosensory genes. The total RNA of the 18 fifth instar larval head, thoracic, and abdominal teguments was extracted following the methods described above. NanoDrop 2008 and agarose gel electrophoresis examined the density and quality of the RNA. cDNA was synthesized from the total RNA, using the PrimeScriptRT Reagent Kit with gDNA Eraser to remove gDNA (No. RR047A; TaKaRa, Shiga, Japan). Gene-specific primers were designed using Primer 3 (http://bioinfo.ut.ee/primer3-0.4.0/) (Supplementary Material 3). Eighteen SRNAs were identified, evaluated, and selected as a reference gene for qPCR (Chen and Hu, in press). A PCR analysis was conducted using the Roche LIGHT CYCLE 480II (USA). SYBRPremixExTaq™ II (No. RR820A; TaKaRa) was used for the PCR reaction under three-step amplification. Each PCR reaction was conducted in a 25-μl reaction mixture containing 12.5 μl of SYBR Premix Ex Taq II, 1 μl of each primer (10 mM), 2 μl of sample cDNA, and 8.5 μl of dH2O. The RT-qPCR cycling parameters were as follows: 95°C for 30 s, followed by 40 cycles of 95°C for 5 s, 60°C for 30 s, and 65°C to 95°C in increments of 0.5°C for 5 s to generate the melting curves. To examine reproducibility, each qPCR reaction for each tissue was performed in three biological replicates (each replicate with six larvae) and three technical replicates. Negative controls without either template were included in each experiment. RocheLIGHT CYCLE 480II was used to normalize the expression based on ΔΔCq values, with GOBP3 in the head as control samples, and the 2^−ΔΔCT^ method was used (Livak and Schmittgen, 2001). Before comparative analyses, the normal distribution and equal variance tests were examined, and all taken logarithm data followed a normal distribution and with equal variances. The comparative analyses for every gene among six tissue types were assessed by a one-way nested analysis of variance (ANOVA), followed by Tukey's honestly significance difference (HSD) tests implemented in SPSS Statistics 18.0. Values are presented as means ± SE.

## Results

### Transcriptome Sequencing and Sequence Assembly

**Figure 2:**
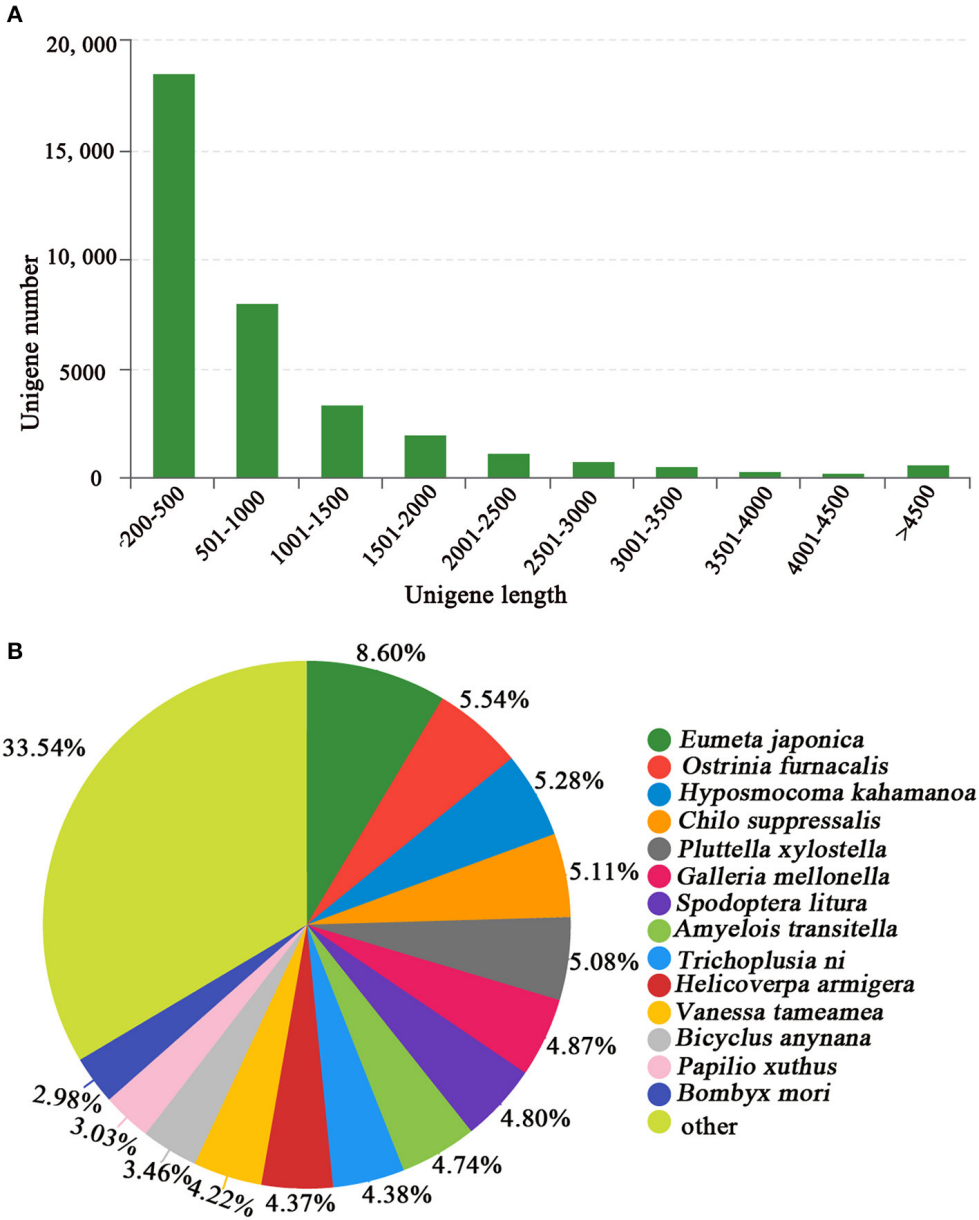
**(A)** Length distribution of *E. signifer* unigenes and **(B)** BLASTx comparison of unigenes in the *E. signifer* transcriptome with those of other species.

We generated 53 million raw reads from a cDNA library derived from the tegument of *E*. *signifer* larvae, with q20 and q30 scores for 97.84% and 93.74% of the reads severally. The larval head transcriptome yielded 51 million raw reads, with q20 and q30 scores for 97.83% and 93.65% of the reads, respectively. After trimming the adapters, removing low-quality raw sequences, using Trimmomatic, blending the head and tegument sequences, splicing, and assembly (using Trinity), we obtained 44,104 transcripts, with an N_50_ of 1,707 bp, an average length of 986 bp, and a maximum length of 56,111 bp (Figure 2A). The *E*. *signifer* raw reads have been deposited in the NCBI Sequence Read Archive database under GenBank accession number PRJNA713545.

### Homology Analysis and Gene Ontology Annotation

**Figure 3:**
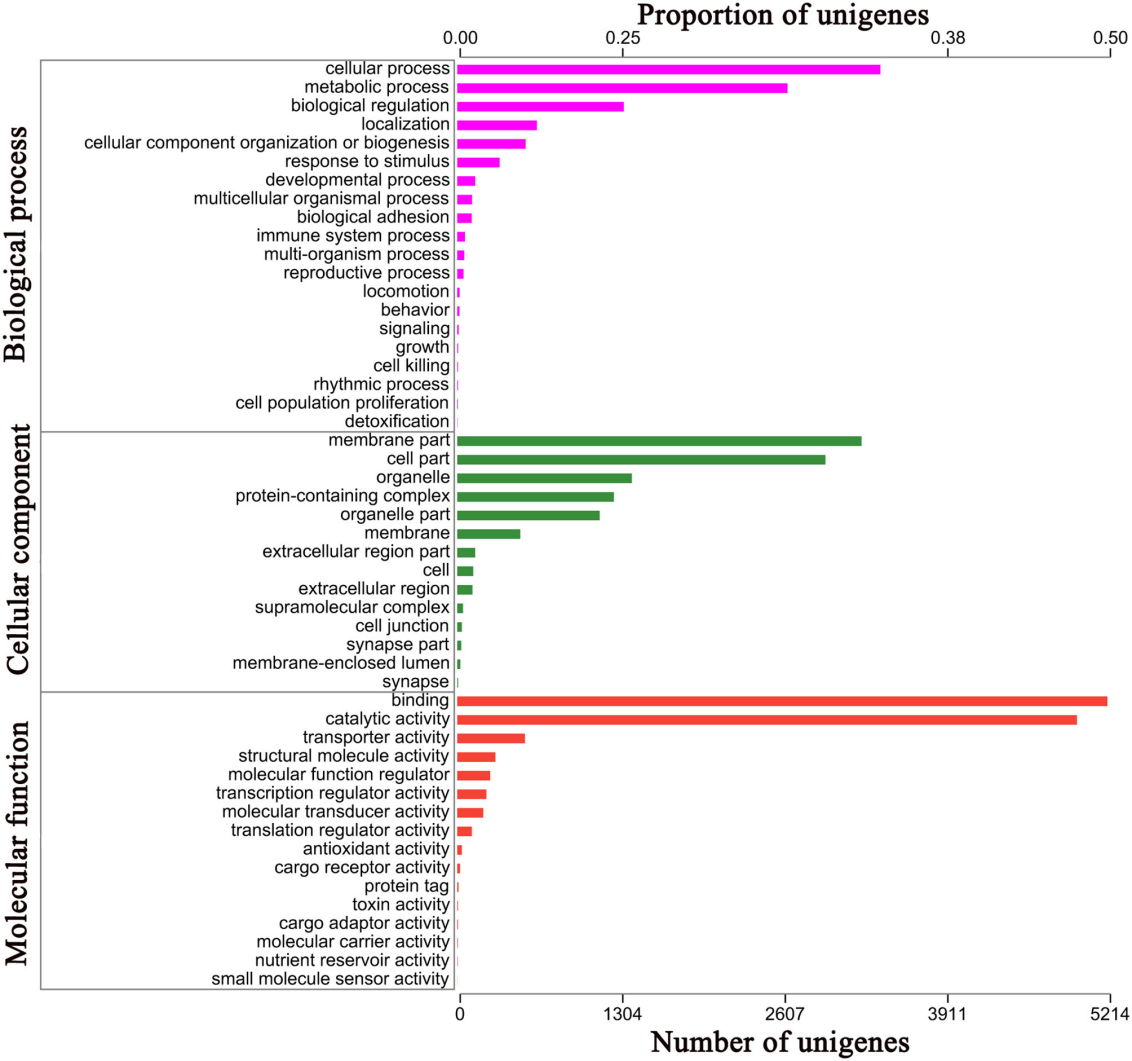
Gene Ontology (GO) annotation results GO analysis of 10,177 genes in *E. signifier* transcriptome, according to their involvement in biological processes, cellular component and molecular function.

For 41.17% of the transcripts, we obtained matches with entries in the NCBI non-redundant protein database, using BLASTx with an E-value cutoff of 1e^−5^. The most frequent sequence matches were with *Eumeta japonica* (6.06%), followed by *Ostrinia furnacalis* (5.77%) and *Hyposmocoma kahamanoa* (5.60%) (Figure 2B). We used gene ontology (GO) annotations to classify the 10,177 transcripts into functional groups with BLAST2GO, with *p*-values calculated from the hypergeometric distribution test and an E-value threshold of <1 × 10^−5^. In the *E*. *signifer* transcriptome, molecular functions accounted for 36.75% of the GO annotations, followed by cellular components (33.96%) and biological processes (29.29%). In the molecular function category, the terms binding, catalytic activity, and transporter activity had the highest representation. In the biological process category, the terms cellular process, metabolic process, and biological regulation were most frequent. Membrane part, cell part, and organelle were the most common cellular component terms (Figure 3).

### Olfactory Proteins

We identified 15 transcripts-encoding putative OBPs in *E*. *signifer*, of which six were general OBPs (GOBPs). According to the FPKM value of the unigenes, EsigGOBP3 and EsigOBP7 were expressed less strongly in the head, and ten OBPs were not expressed in the tegument (Table 1). We identified six transcript-encoding putative CSPs, of which four were expressed more strongly in the head, whereas CSP1 and CSP2 were expressed more strongly in the tegument (Table 1). One transcript encoded a putative SNMP and was strongly expressed in the tegument (Table 1). Two identified ORs were strongly expressed in the tegument (Table 1). One transcript encoding a putative GR was strongly expressed in the head. We identified 14 IRs, of which EsigIR1, EsigIR75p-1, EsigIR40a-1, EsigIR93a-1, and EsigIR5 were more strongly expressed in the tegument, whereas the others were expressed in the head (Supplementary Material 4).

**Table 1:** Best blastx hits for odorant-binding proteins (OBPs), chemosensory proteins (CSPs), odorant receptors (ORs), gustatory receptors (GRs), and sensory neuron membrane proteins (SNMPs) of *Endoclita signifier*.

**Name**	**Nr description**	**Species**	**Acc. NO**.	**Tegument FPKM**	**Head FPKM**	**Tegument vs. Head**
EsigOBP1	Odorant binding protein LOC100307012 precursor	*Bombyx mori*	NP_001159621.1	0	1.39	Down
EsigOBP2	Odorant binding protein 7	*Grapholita molesta*	AVZ44706.1	0	4.32	Down
EsigOBP3	Odorant binding protein LOC100307012 precursor	*Bombyx mori*	NP_001159621.1	0	8	Down
EsigOBP4	Odorant binding protein LOC100307012 precursor	*Bombyx mori*	NP_001159621.1	0	4.04	Down
EsigOBP5	Odorant binding protein	*Eogystia hippophaecolus*	AOG12872.1	0	2.97	Down
EsigGOBP1	General odorant-binding protein 70-like	*Amyelois transitella*	XP_013201142.1	0	2.97	Down
EsigGOBP2	General odorant-binding protein 56d-like	*Hyposmocoma kahamanoa*	XP_026319368.1	0.45	4.87	Down
EsigGOBP3	General odorant-binding protein 83a-like	*Plutella xylostella*	XP_011554700.1	1.48	0.39	Up
EsigGOBP4	General odorant-binding protein 19d-like	*Papilio xuthus*	XP_013173035.1	0	4.47	Down
EsigGOBP5	General odorant-binding protein 19d	*Eumeta japonica*	GBP31818.1	0	4.98	Down
EsigGOBP6	General odorant-binding protein 28a-like	*Hyposmocoma kahamanoa*	XP_026330999.1	0	3.07	Down
EsigOBP6	Odorant-binding protein 16	*Ectropis obliqua*	ALS03864.1	18.31	107.65	Down
EsigOBP7	Putative odorant-binding protein A10 isoform X2	*Zeugodacus cucurbitae*	XP_011177223.1	101.91	98.21	Down
EsigOBP8	Odorant binding preotein	*Conogethes punctiferalis*	APG32543.1	0.07	30.95	Down
EsigGOBP7	General odorant-binding protein 1	*Athetis dissimilis*	ALJ93806.1	0	13.48	Down
EsigCSP1	Chemosensory protein 10	*Carposina sasakii*	AYD42214.1	1.82	0.52	Up
EsigCSP2	Chemosensory protein 24	*Cnaphalocrocis medinalis*	ALT31606.1	181.82	2.78	Up
EsigCSP3	Chemosensory protein 5	*Agrotis ipsilon*	AGR39575.1	9.65	10.58	Down
EsigCSP4	Chemosensory protein CSP14	*Lobesia botrana*	AXF48711.1	3.16	9.35	Down
EsigCSP5	Chemosensory protein 5	*Empoasca onukii*	AWC68022.1	0	71.07	Down
EsigCSP6	Chemosensory protein	*Cnaphalocrocis medinalis*	AIX97837.1	10.33	218.45	Down
EsigOR1	Odorant receptor 28, partial	*Locusta migratoria*	ALD51442.1	4.17	0	Up
EsigOR2	Odorant receptor OR4	*Rhyacophila nubila*	AYN64394.1	0.98	0.94	Up
EsigGR1	Gustatory receptor	*Eogystia hippophaecolus*	AOG12970.1	17.74	76.58	Down
EsigSNMP1	Sensory neuron membrane protein 2 isoform X1	*Neodiprion lecontei*	XP_015517411.1	1.87	0.16	Up

### Olfactory Protein Expression Profiles

**Figure 4:**
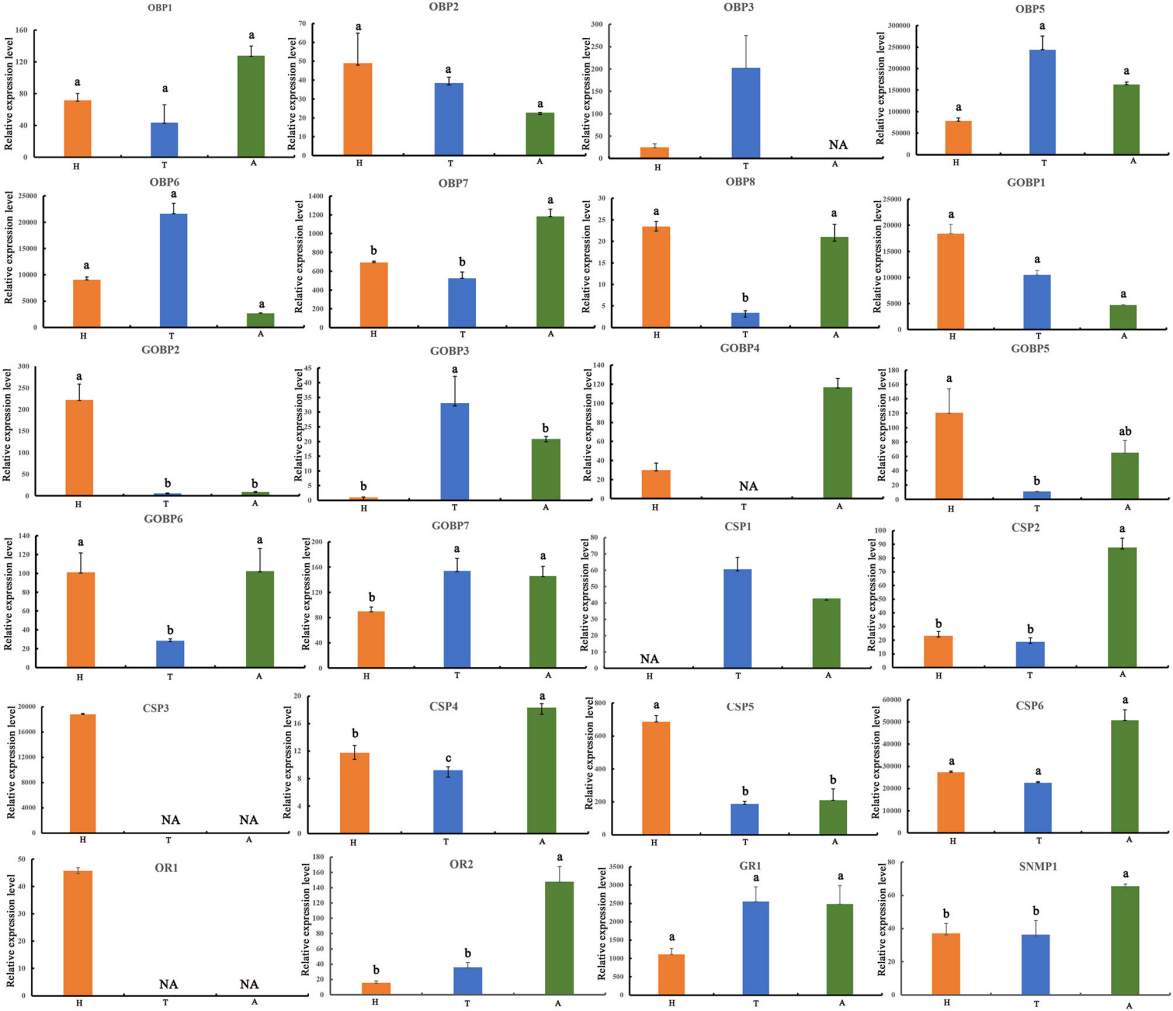
Expression profiles of *E. signifer* olfactory proteins in three tissues. H, head; T, thoracic cuticle; A, abdominal cuticle, 18S was used as the reference gene to normalize target gene expression. The standard errors are represented by the error bars, different lowercase letters (a,b,c) above the bars denote significant differences at *p* < 0.05.

We characterized the expression profiles of all the OBPs, CSPs, ORs, GRs, and SNMPs in the head, thoracic, and abdominal tegument of fifth *E*. *signifier* larvae. Of the OBPs, EsigOBP5 was the most strongly expressed OBPs in all larval tissues (10- to 1000-fold higher values). EsigOBP2, EsigOBP8, EsigGOBP1, EsigGOBP2, and EsigGOBP5 were most strongly expressed in the head, and EsigOBP8 and EsigGOBP5 were expressed at significantly lower levels in the thorax than in the head. EsigGOBP2 exhibited significant head-biased expression. In non-olfactory tissues, EsigOBP3, EsigOBP5, EsigOBP6, EsigGOBP3, and EsigGOBP7 were most highly expressed in the thoracic tegument, with a significant thorax-biased expression of EsigGOBP3. EsigGOBP7 was expressed at significantly higher levels in the thorax and abdomen than in the head. EsigOBP1, EsigOBP7, EsigGOBP4, and EsigGOBP6 were most strongly expressed in the abdominal tegument, especially EsigOBP7. Significantly, more EsigGOBP6 was expressed in the head and abdomen than in the thorax (Figure 4). Of the CSPs, EsigCSP3 and EsigCSP6 were the most strongly expressed CSPs, with levels 100- to 1,000-fold of other CSPs, and EsigCSP3 was only expressed in the head, but EsigCSP6 was strongly expressed in all larval tissues. EsigCSP5 and EsigCSP3 were most strongly expressed in the head, especially EsigCSP5. The expression of EsigCSP2, EsigCSP4, and EsigCSP6 was highest in the abdomen, especially for EsigCSP2 and EsigCSP4. EsigCSP4 expression differed significantly from the others in the three tissues, and EsigCSP1 was highly expressed in the thorax (Figure 4). EsigGR1 was expressed most strongly in the abdomen (Figure 4). EsigOR1 was expressed only in the head, whereas, significantly, more EsigOR2 was expressed in the abdomen than in other tissues. We also detected significantly higher EsigSNMP1 expression in the abdomen compared with the other tissues (Figure 4).

### Phylogenetic Analysis of OBPs and CSPs

**Figure 5:**
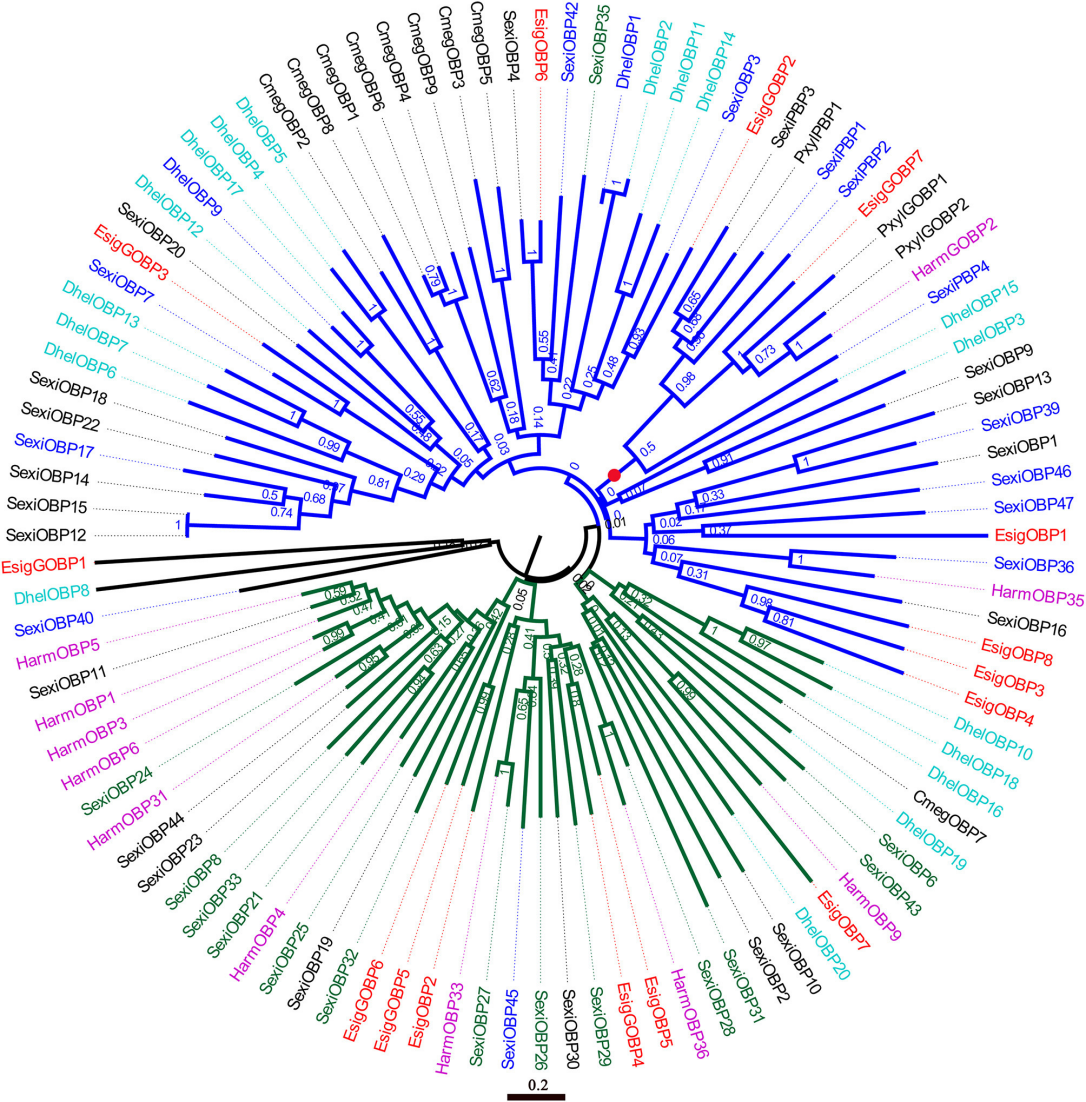
Neighbor-joining phylogenetic tree of odorant-binding proteins (OBPs). The NJ phylogenetic analysis of OBPs of *E. signifier* (EsigOBP, red) was performed with reference OBPs of *D. helophoroides* (indigo blue), *C. megacephala* (black), *P. xylostella* (black), *S. exigua* (blue), *H. armigera* (purple). Green OBPs/GOBPs of *P. xylostella* showed only larvae expression. The stability of the nodes was assessed by bootstrap analysis with 1,000 replications. The scale bar represents 0.2 substitutions per site.

**Figure 6:**
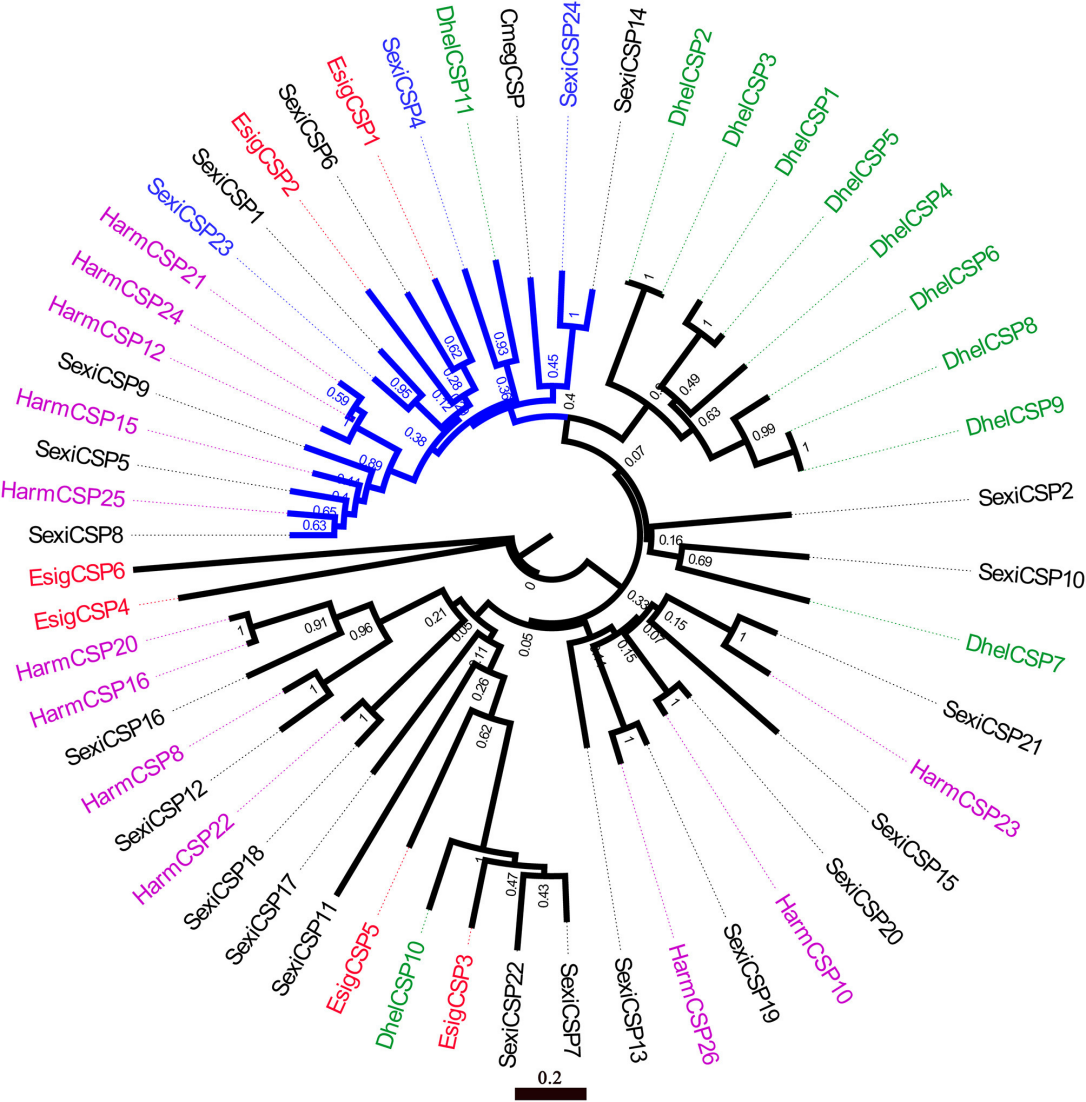
Neighbor-joining phylogenetic tree of chemosensory proteins (CSPs). The NJ phylogenetic analysis of CSPs of *E. signifier* (EsigCSP, red) was performed with reference CSPs of *D. helophoroides* (green), *C. megacephala* (black), *S. exigua* (black), *H. armigera* (purple) and *E. signifier*. Blue CSPs of *P. xylostella* showed no larval expression. The stability of the nodes was assessed by bootstrap analysis with 1,000 replications, and only bootstrap values ≥0.6 are shown at the corresponding nodes. The scale bar represents 0.2 substitutions per site.

In the phylogenetic tree of OBPs (Figure 5), the no-expression clade (blue) included EsigGOBP3, EsigOBP6, EsigGOBP2, EsigGOBP7, EsigOBP1, EsigOBP3, EsigOBP4, and EsigOBP8. The OBPs not expressed in larvae included SexiOBP3, SexiOBP7, SexiOBP9, SexiOBP17, SexiOBP36, SexiOBP39, SexiOBP42, -] SexiOBP46, SexiOBP47, SexiPBP1, SexiPBP2, SexiPBP4, DhelOBP1, and DhelOBP9. The expression clade (green) included EsigGOBP6, EsigGOBP5, EsigOBP2, EsigGOBP4, EsigOBP5, EsigOBP7, with SexiOBP8, SexiOBP21, SexiOBP24, SexiOBP25, SexiOBP26, SexiOBP27, SexiOBP28, SexiOBP29, SexiOBP31, SexiOBP32, SexiOBP33, and SexiOBP6 expressed only in larvae. The PBP clade (red circle) contained EsigGOBP7; four PBPs of *S*. *exigua*; PBP1, GOBP1, and GOBP2 of *P*. *xylostella*; and HarmGOBP2 (Figure 5). In the phylogenetic tree with CSPs, the no-larval-expression clade (blue) included EsigCSP1, EsigCSP2, and SexiCSP4, SexiCSP23, and SexiCSP24 (Figure 6).

## Discussion

Larval survival contributes to its host selection. From the head and tegument transcriptomes, we identified 39 olfactory proteins, including 15 OBPs, 6 CSPs, 2 ORs, 1 e GR, 1 SNMP, and 14 IRs. This is the first report of the separate larval head and tegument transcriptomes in Hepialidae. The number of olfactory proteins identified in *E*. *signifer* is less than that reported for most adult antennae transcriptomes, such as *Conogethe spinicolalis* (Jing et al., 2020). Additionally, larvae tend to have a shorter squirm range and a less complicated survival environment than adults and may consequently express a smaller set of olfactory genes than adults, as observed for *Spodoptera littoralis* (Poivet et al., 2013). The number of olfactory proteins in *E*. *signifier* is considerably fewer than the 26 OBPs and 21 CSPs identified in the transcriptomes of *H. armigera* larval antennae and mouthparts (Chang et al., 2017); the 20 OBPs, 11 CSPs, 9 ORs, 11 IRs, 7 GRs, and 4 SNMPs in the transcriptomes of newly hatched *Dastarcushelophoroides* larvae (Li et al., 2020); and the 58 ORs, 20 GRs, and 21 IRs in the transcriptomes of the antennae of males and females and the head tissue of neonates of *Cydia pomonella* (Walker et al., 2016). This reflects the scarcity of olfactory proteins of the original Lepidoptera group in the NCBI database, such as Hepialidae, resulting in less annotation of olfactory proteins in *E*. *signifer*.

According to the OBP expression profiles in larvae, distinct no-larval-expression or larval-specific-expression clades were apparent in the neighbor-joining tree based on OBPs, with EsigGOBP3, EsigOBP6, EsigGOBP2, EsigGOBP7, EsigOBP1, EsigOBP3, EsigOBP4, and EsigOBP8 placed in the no-larval-expression clade. EsigCSP1 and EsigCSP2 belonged to the no-larval-expression clade in the CSP phylogenetic tree, and EsigCSP1 was not expressed in the larval head. However, EsigGOBP4, EsigGOBP5, EsigGOBP6, EsigOBP2, EsigOBP5, and EsigOBP7 were placed in the larva-specific-expression clade, and EsigOBP5 and EsigOBP7 were highly expressed in larvae compared with the other genes. The small number of proteins known to be specifically expressed in larvae may cause false positives in the larva-specific clade. EsigGOBP7 was placed in the PBP clade, suggesting that it is an *E*. *signifer* PBP and that PBPs expressed in larvae function as sex pheromones binding (Zielonka et al., 2016).

The expression profile of olfactory proteins in *E*. *signifer* larvae showed that all were expressed in at least one tissue, verifying the olfactory proteins identified in the head and tegument transcriptomes. EsigCSP6, EsigOBP5, EsigGOBP1, EsigOBP6, EsigOBP7, and EsigGR1 were highly expressed in all tissues of the fifth instar larvae, and the high expression of olfactory proteins indicates that *E*. *signifer* larvae require many olfactory proteins to support olfactory recognition, especially the transfer from soil to standing trees. Additionally, we observed the expression in all tissues, except the head, thorax, and abdomen of borers, for some proteins with olfactory functions, while some olfactory proteins with no olfactory functions, such as OcomCSP12 of *Ophraella communa*, are expressed in female ovaries, and silencing of OcomCSP12 results in significantly reduced ovipositing by females (Ma et al., 2019). Eating is the main behavior in larvae, which may explain why EsigGR1 exhibited the highest expression among all tissues, and EsigGR1 may correlate with the feeding habits of *E*. *signifer* larvae and reflect gustatory preferences.

The head is the center of sensation. EsigCSP3 was strongly expressed only in the head, and CSPs are known to contribute to mediating responses to plant volatility in *Mythimna separata* (Younas et al., 2018) and *Nilaparvata lugens* (Waris et al., 2020). EsigCSP3 might play key roles in the process of sensing *Eucalyptus*-derived compounds in *E*. *signifer* larvae. Importantly, eight genes were expressed most strongly in the head (EsigOBP2, EsigOBP8, EsigGOBP1, EsigGOBP2, EsigGOBP5, EsigCSP3, EsigCSP5, and EsigOR1), with the expression of EsigGOBP2, EsigCSP5, and EsigOR1 biased to the larval head, consistent with the observation of 50 *S*. *exigua* ORs expressed in larval heads (Llopis-Gimenez et al., 2020) and larvae (Liu et al., 2015). For *S*. *exigua* OBPs, expression levels are higher in the larval head than in the larval body (Liu et al., 2015). In the larval head, many *S*. *littoralis* OBPs and CSPs exhibit organ-specific transcription in caterpillar antennae and maxillary palps, suggesting the complementary involvement of these two organs in larval chemosensory detection (Poivet et al., 2013). Moreover, *H*. *armigera* expressed more OBPs and CSPs in the larval antennae than in the mouthparts (Chang et al., 2017). Therefore, it is necessary to examine the expression of the eight most strongly expressed olfactory genes in the antennae and mouthparts of *E*. *signifer* to determine their roles in smelling and tasting the odors of the host plant (Jin et al., 2015; Di et al., 2017; Waris et al., 2020).

Furthermore, the expression profiles of olfactory proteins in *S*. *exigua* larvae indicated that they are also expressed in non-olfactory tissues, such as the larval body (Liu et al., 2015; Llopis-Gimenez et al., 2020). In *E*. *signifer* larvae, most olfactory proteins were highly expressed in thoracic and abdominal tegument, consistent with the large number of sensilla on the thorax and abdomen (Hu et al., 2021). This establishes the molecular basis for the head and the other main sensor tissues. Borers need to use non-olfactory tissues, such as the thorax and abdomen, to sense the wood hole environment and adapt to wood hole survival. It is necessary to explore the functions of olfactory proteins that are highly expressed in non-olfactory tissues, such as EsigGOBP3, EsigOBP7, EsigCSP2, EsigOR2, and EsigSNMP1. The patterns of olfactory protein expression during larval development have also been studied. For example, an OBP (Cmeg33593_c0) in *C*. *megacephala* is increasingly expressed from the first to the third instar larval stages, and the larval olfactory protein expression profile indicates that some proteins are expressed only in larvae (Wang et al., 2015). Two binding proteins appear to be larva specific in *S*. *littoralis* (Poivet et al., 2013) and green SexiOBPs in the OBP phylogenetic tree (Llopis-Gimenez et al., 2020), as well as six OBPs and four CSPs are larval tissue specific in *H*. *armigera* (Chang et al., 2017). More larval olfactory proteins are expressed in both larvae and adults of other species, as demonstrated for several *C*. *pomonella* ORs that exhibit sex-biased expression in adults, as well as larva-enriched transcription (Walker et al., 2016). Based on these results, the expression profile of *E*. *signifer* olfactory proteins should be further explored in the antennae and mouthparts and at various developmental stages.

## Conclusion

We identified 39 olfactory proteins in *E*. *signifer* larvae, with EsigOBP2, EsigOBP8, EsigGOBP1, EsigGOBP2, EsigGOBP5, EsigCSP3, EsigCSP5, and EsigOR1 expressed most strongly in the head. CSP3 was expressed only in the head, where it plays key roles in sensing *Eucalyptus-*derived compounds, whereas EsigGOBP2, EsigCSP5, and EsigOR1 exhibited biased expression. EsigGR1 exhibited the highest expression among all tissues, which may correlate with the feeding habits of *E. signifer* larvae based on gustatory preferences. The functions of EsigGR1 and EsigCSP3 in larval olfactory and gustatory recognition should be explored further. Most olfactory proteins were highly expressed in thoracic and abdominal teguments, establishing the molecular basis for the head as the center of sensation and explaining how borers use the thorax and abdomen to sense the wood hole environment.

## Data Availability Statement

The datasets presented in this study can be found in online repositories. The names of the repository/repositories and accession number(s) can be found below: https://www.ncbi.nlm.nih.gov/, PRJNA713545.

## Author Contributions

XYZ carried out the molecular genetic studies, performed the sequence alignment, experiments and drafted the manuscript. XHY, HXM, and XML collected all samples and participated in some experiments. PH and ZDY designed and conceived of the study. PH also helped to draft the manuscript. All authors read and approved the final manuscript.

## Conflict of Interest

The authors declare that the research was conducted in the absence of any commercial or financial relationships that could be construed as a potential conflict of interest.
