# Supplementary material for: Olfactory Proteins and Their Expression Profiles in the Eucalyptus Pest Endoclita signifier Larvae
Source: Front Physiol. 2021 Jul 19;12:682537. doi: 10.3389/fphys.2021.682537 (PMC8327093; doi:10.3389/fphys.2021.682537)
Supplement: Supplementary file 1 [file Table_1.DOCX]

Olfactory proteins and their expression profiles in the Eucalyptus pest *Endoclita signifie*r larvae

Xiaoyu Zhang^2^, Xiuhao Yang^3^, Hongxuan Ma^4^, Xiumei Liu^4^, Zhende Yang^1^, Ping Hu^1, 2*^

**Supplementary file 1 Nucleic acid sequences of all candidate chemosensory proteins identified in *Endoclita signifier* transcriptome**

>EsigCSP1

ATGAGTCCATTGACAGTGGCGTGCCTACTAGCGGCAACATGTTTTGTGCACGCGGCCCCG

GAACACTATACGAGCAAGTACGATGGCATCAATTTGGATGAGATCCTCGCCAACCGGCGC

CTCTTCGTGCCCTACATCAAGTGCATCCTCGACCAGGGCAGGTGCACTGCCGAGGGCAAG

GAGCTTAAAGCACACATCAGCGATGCGTTGCAGACGGGCTGTAGCAGCTGTACGGCGACG

CAGCGCAAGGGTGCTCACAAGGTCATCGGACACCTCATCCACCACGAAGGCGAGTTCTGG

CAGCAGCTGGTTGACAAGTACGACAAGACGCGCCAGTACACGCGCAAGTACGAGCACGAG

CTCAAGAGCATTGCCGCG

>EsigCSP2

ATGTTACTGCGTAATAGAGTGGGTGTGGTCGCGCTGGTGGTGCTGGCGGCGGCGGCGGTG

GCCACGGCGGCGCAGACCCGCTACTCGGAGCGCTACGACCAGGTGAACGTGGGCGAGGTG

CTCGCCAACCGCCGCCTCTTCACGCCCTTCGTCAACTGCATCATGGACCGCGGCCGCTGC

ACCGCACAGGGCCGCGAGCTAAAGAACCACCTGGCGGACGCGCTGCAGAGCGGGTGCGCG

CGCTGCTCGGCCAAGCAGCAGGCGGGCGCCACGCTCGTCATCCAGCACCTGATCCACCGC

GAGCCGGCCGTGTGGCGTGAGCTGACGCGCAAGTACGACCCGTACGCCATCTACCGCAAC

AAGTACGAGGCCTTCGCCGCCTCTAAAGGCATCACGCTGCCCAAACACTAA

>EsigCSP3

ATGCAGCCCACAATTCTGTTTATCTGCTGCGTCGTCATCGCTGCATCAGCTTCAGCGGCT

GTAACACCACGTCCCGCCGTATCCGATACAGCACTTGACAACGCGCTGCAGGACAAGAGA

TACCTCATGAGACAGCTTAAATGCGCTTTGGGTGAGGCACCTTGTGATCCCGTTGGGAGA

AGATTGAAAAGCTTAGCTCCATTGGTACTTAGAGGTTCGTGTCCTCAGTGCACGCCGCAG

GAGATGAAGCAGATCCAGAGAACACTGGGACACGTACAGAGACATTATCCCAAAGAATGG

GCTAAGATGCTCAAACAGTATGCCGGAAAGTAA

>EsigCSP4

GCGCCCTACGTGGCACGACAGGCCGCGCCGCCGGCCGCACCGCCCGCCGCATTGCCGGCC

CTCAATACGCTCGACACCGTCGACGCTCGGCGCGAGAGTAAGCTGGCCCGACTACGCCTG

GAGGAGAGCATGGTACCGCTCTCGCCGCTCGACACCAAGTGCCTCACATACAAGACGGAC

GAACTCTGTAGCGGCCACATGAAAAGTGTCAAGGCGACGCTGGTCTCGGCGTTGCAGAAC

GGCTGCGCGTCGTGCGGGGCGGGCGAGCGTGCGGCCGCGGGCGCCGTCGCCGCCGCACTC

CTGTTCCACCATCCCCACGTGTGGAAGCTGCTGGTGCTGCGCCACGGTCGTGACAACGCC

AGGTACATTTGA

>EsigCSP5

GCGAAGGGCCCTTGCGATCCCTTCGCCAAACACTTGCGAGACAACTTGTTGGAGGTGGCA

GGGACAGAGTGCGAGGTATGCAGTCCGAAGCAGGCGGAGCAGTACGAGAAGACGATGATT

GCTATACACAATCTGTATCCCAAGGAAGCCGAGAAAATTTATAACAAGTATGACCCGGAG

ATCAAGTGCCGCACTGCCATTAAAGCTTTGATAGAG

>EsigCSP6

ATGGCCTTAGAGACGTTTTCACTCGAAAATCCACTTTTCCGTACCTATCTTGTTTACTCA

TCAATTCTCGTTTTAAAAATGTTGATCATGTCTGTTTTGACCGCACGGCAGCGTTTTAGG

AAAGGGGTATTTGCCAATGCCGAAGACGCTAAAACTCCAAAAGCAAAGGTCAAGTACGAT

GATGTTGACGTGGAACGTGTTCGAAGAGCTCATCAGAATGACTTGGAAAATATTCCAATC

TTCCTCGTGATAACTTCCATGTATATACTGATTGATCCACCAGTATTCCTAGCGACCAAC

CTGATCAGGTTGTTCACAGCAGGACGAGTGCTTCACACTTTGGTATATGCTGTCTGTCCC

GTTCCACAGCCTGCACGTGCTTTGGCTTGGGCTGCTGGTTATTTTACCACTATTTTTATG

GCCACTTCCGTATTTATAAAATTTATATCTGATTTGTAA

>EsigOBP1

AGCGAGAAAAATGCCGACTGGGCGGCCGTAACGACAGAAATAAAGGAGAAATGCTTTAGG

AGTGAGCTTTTCGAACGCGGACCTCCGGTCTGTCAGCCGATGAAGCTTACTTTCTGCATC

TCCCGAGTATTTCTTATGAATTGTCCTGCTAGCCATTGGAACGACACAACTGTTTGCAAT

GATATGAAAAAGTACATGACTGACTGTTTCAATAAGCGTCATCATTGA

> EsigOBP2

ATGTTGAAATACTTGGTACCGGTTCTAGTCCTGGCCCTCTTCGCCGGTGTCGAGGCAATG

ACTAAGGAAGAAATGAAAGAAATGTTTATGGAGATCGTGTCTGGCTGCCGCGATGATAAC

CCTGTCTCGGACGAGGAGATCACCCAACTCCACCAGAAGCAGCTGCCTGACGGCGGCAAC

GCCAAGTGTTTGCTCAACTGTGTCGTTTCCACTATTGGTATTATGGAAAATGGTGTGCTG

AGCGAGGACGCGGCGCACGCGCTTGCGGAAAAGCACTTTGAAGATGCTGAAGAACTGAGC

GCT

> EsigOBP 3

ATGTTCGACGCTGTCTCCAGAGTGGTTTTAGTGCTAGTAGTTTATGCAAAACTGGATTGT

GGTGCGGCTGATATATGTGCAACACCACCTGCGATAATCAATCCTCGCGACTGCTGTGAA

TATCCTTGTTTGTTTGACAAGTCGATTCTCGAGGAATGCGGCATCGTGGAAACGGATAAA

GGTCTCAAAGAAACTCCAACATGCGAACAACTGACGTGCTTCTTTAAGAAAACCAACATG

ATGACGGATGACCAGAATATAAATGTGGAAGGTGGAAAAGCCCACGTAGCCAAGTGGCTC

GCCAGCCACCCCGAGTGGGCGGAACAAAGTGACTTAGCTAACAATTGTTTCTCCGAAGCC

GCCTTGAAGGCGGGACCTCCGGGGATGTGTGATCCGTTAAAGATAACTATCTGCGTCAAT

AAAGTATTGTTAAAGTTCTGTCCTGCCAAGTTCTGGAAGTCATCTGACCAATGCGAAAAC

TTGAAAGCCTAC

> EsigOBP 4

ATGTTCGCCACTGTTTCCAGAGTGGTATTAACATTTGTTATTTTTGCATTACAGAAGCGA

AACTGCGATGCTGCTGATATGTGTGCAACACCACCTCCGATGTTAAATCCTCGCGAATGC

TGTGGATTTCCCCCGTTATTCGATCAATCGAATCTTGATGAATGCGGTGCTCCAGGCTCT

GCTAAAAATGAAATGGGGCTTCCAAAATGCGACCATTTGGACTGCATTTTAGAAAAAGAG

AACTTATTAACAAATGACCATAAAATGAATATTGAAGATGGAAAAGCCCATTTAACCAAG

TGGTTTGCAAACCACCCAGAATGGGCAAACCACAGCGATGATGTAGTCAATAGATGCTTC

TCCGAAGACACTTTGAAATCGGGACCTCCGGACATGTGTCATGCATTGAAGGTCATGCTA

TGTATTGCTAAATCGTTTTTAATATCTTGTCCTGCCAGCTACTGGCATCCATCAGAG

> EsigOBP 5

CTGCAGCAGTATAAGATACCAGCAACAGGAAATGGCAAATGTTTACTGGCTTGTGCTTGC

AGGAAAATCGGTGCTATAAATGACAAAGGCATGTTCGATACGGCTAATGGAATTAAAATT

GGTAAAAACTTATATGGAGCTGAAGGTGGAAAGGCAGATAAAGCAAAAGATCTCGTTGAC

ACTTGCAAAGGCGTAAATGATGAGCCAGTGACAGATGGTGAAAAGGGTTGCGATCGAGCT

GCACTTCTGGTGAAATGCATAGTCAAGAATGCAAAGAAGCTTGGCTTTAAAATCTAA

>EsigGOBP1

AATCTTCAAGTACTTTCATCTAACAGACAGCCCCCAGTCCCCGGCGGCTCCACTATTCTT

GGCAAAGGTAGCAGCCATGGTTACGGCGGAGCAGGGTGTGCTAGAAATATCTGGCAGAGG

CCCGGCTACCAACACCCATGATCTTTAGACCTGGCAACATATAACGCACGCACATTGAGG

ACTGACGAGAAGTTGATTGAGCTGGAAGAAGTAGTAGATAAGTTGCGCTGGGATGTTATA

GGATTATCAGAAGTCCGAAGAGAGGGGGAGGACACGATGTTTCTCAATTCCGGCAACTTG

TTCTACTACCGGGAGGGCGACCAGCTGTCCCAACAAGGTGTCGGGTTTCTTGTCCACAAG

TCTCTCGTCAATAACATTGTGAAGATCGAAAGCGTTTCGAGCAGAGTGGCGTACCTTATA

CTCAGAATCACCAAATGGTATTCGCTGAAGGTGATACAGGTATACGCCCCGCACTCGAAA

CGCCCGGACGATGAAGTGGAGGCTATGTATGACGACATTGCAAATGCAATGCATGCCTCG

AAAACATACTTCACAGTGGTGATGGGAAACTTTAACGCGCGGCTAGGCATGGGAAACAGC

GATGAACTGAGAGTGGGGCCATTTGGATTTGGACAGTGAAACCAAAGGGGCCAGCGATTG

GCCGACTTCATGGAAAAGGAGGGACTCTTTGCGATGAACACCTTCTTCAAGAAGTGGCCG

TCCACGAAATGGACGTGGCTACATCCCGCCGGTTACAAGTACGAGATGGACTTCATCATG

ACGACCAAGAAGCAAATATTCAATGATGTCTCAGTGATCAATAGCGTCAAAACTGGCAGT

GATTACCGTCTAATGAGAGGCTCATTGAATATCGACGTTAAGCTAAAGAGGTCCCGTCTA

ATGAAGTCTACTCTTCATCCAAATCCTGTCCAAATTCAAAGCCCCAAGAGCTTTCAGCTA

GAACTCCAAAATCGCTTCCAGTGCCTGACTGAATGCGAGACAGTGGACGAGCTGAACAAC

GGGCTGGTGGAAATTGTCCACGCGGGTGGGTCGGAATTCTTTAGGACCCATCGCAAAAAT

GGAACTTGTAAACTCTCTGAGCACACTCTTAAACTCATGGCAGAGCGAAACAAAATGAAA

CTGCAGTCTTCAGCAGATATGTCGAAATATAGCCTGCTCAATAGGCAGATTTCCAAG

> EsigGOBP2

ATGAAGTTTTACATAATAGTGCTAACTGCTGCACTTGCAGTTGCATCGGCGGATGAAACC

CAGCTAACAGACGAGCAGAAGGCGCGAGTTCTGCAGTTCTCGGCGGACTGCATACGCGAA

ACAGGCGTCAAACCTGAGCTAGTATCCGAAGGGAAAAATGGAAAATACTCAGACGACGAG

GCTTTCAAGAAATTCACTTACTGTTTTTTCAAAAAATCCGGAATAATTAATTCGGAGGAT

GAGCTAGACGTCGACGTTGCGTTATCCAAGCTACCCTCCGCAGTGGACAAGGAGTCAATA

AGAAAAGTCCTAGAAGAATGCAAACAGAAGAAAGGAAGTAATCCTGCCGATACGGCCTTC

GAAGTCTTTAAGTGCTACAAAGCGGCTACGCCCACGGATATAGATACATTTTAA

> EsigGOBP3

GGTGCGTTGGGAATTTGTCTACTTGGACACCACCATCATTGTCACCTTGAATATATACTG

ACCAAAGAATTTCCCGAGGAGATTAAGGAGATCGTCGACCACCTACACAGAACTTGCGTC

GACGTAACGGGAGTCAGCGAAGAGGACATCGCCAATTGCGAGCGCGGCGTCTTCAAGGAG

GATAAAAAGCTCAAATGCTACATGTTTTGCCTCATGGAGGAGGCGAGTTTTGCGGAAGAA

GACGGCACCGTCGACTACGACATGCTGACAGCCATGATTCCCGAACAGTGGAGGGAGAAG

GTGACGTCTATAGTAATGGCGTGCAAGTATTTAGACACGCCTGATAAAGACAAGTGCGAG

AAGAC

> EsigGOBP4

ATGATTGGAGTGCAATTTTTGTCTGCAGTTTGTATAACGCTCGTGGCATCGGTTCAATGT

TACTCAGATGAGGAAGTAAAGATGAAGTACACGCAAGTATTCGTGGAGTGCGCAGCGGGC

ACACCCGTCTCCAAGGAGGACATAGCGATGTTGGAGAAACAGGAGTTACCGGAAAACGAC

AATGTCAAGTGCGCTCTAGCCTGCGTCTACAGGAAGTTCAATGCTATGGATGACAATGGT

GTGTTCACTCCCGCACTGAGTGAGAAAGCTGGACGCGAGCTGTATGCTGGTGCTCCCGAA

GAAAAATTAACTAAGCTCAAAGAAGTGAGCGAGACCTGCACGAAAGTAAACGACTTAACC

GTAAGTGACGGTGCGAAGGGTTGTGATAGAGCAGGACACATCTTCAAATGCCTCCACGAA

GAAGCTAAGAAGGTGGGCGTCGTAATGTATTAA

> EsigGOBP5

CGCATTTTTTCGAAGAAAATTTTCTTAAAAATGTTGGTTCTCATTGCTTTAATGGCTGCT

GTTGGTGTTGTTCACAGTGAAATGAGTGAAGAATTTAAAGCTGCACAAGAAAATTGCCGG

AAGTCAGTCACGATAACTGACGAAGGTTTTGAATCACTTATTAGTGAAAAGAAGCTGCCA

AGCACCAAGGAAGAGAAATGTTTTTTGGCCTGCCTCTATAAGGAAGTCGGCTGTATGGAT

ACTGACGGGAAGATTTCAGAAGAAGGTATGCTAGCATTCGCTGAGAAAAAATACAAAGAT

GACGCAGAGAAAAAAGAAGGTCTATCGAAGGTTATCAAGATATGCCACAAAGTAAATGAC

GTGGATGTATCCGATGGTAAAGAAGGTTGCGAGAGAGCTGCTCTAAGCCTTGAATGTATG

CTCAAAGAAAATAGTGACATTGTTCTCGCCTTGAAGTGA

> EsigGOBP6

ATGGCTTTCTTCAAGCTTTTCGCGTTTGCTGTTATTGTCGCTGCTATTAACGCCGCTCCC

AGTGACCCGACCGATCAGAAACAGACCTTACAGTCGATTGGCAAAGCATGTCGCGTGAAA

ACTCCGGTAACTGACGAAGAATATCAAGAACTCTTGGATAAAAAGCTTCCTACCACCAAG

GACGCTAAATGTCTTTTGGCCTGCATCTACAAGACTGTAGGCATTCTGGGCGATGATGGG

CTCATTTCCGAGGAAGGTTCCCTAGCCTGCGCAGAAAAGAAGTATAACGACGACCCAGTC

AAAAAGGAGATCCTGTTGGAAGTTGTCAAGAATTGCCATAAAGTGAACTCTGTGGCTGTG

TCCGACGGAACCGACGGCTGTGAACGAGCCACACTGAATCTTGAATGTATGCTCAACGAG

AACAGCGCGATTGTGACCGGATTCGACTAA

> EsigOBP6

ATGAAGTACCTAGTCGTGATCGCAGCTTGCCTCGTCGTGGCCGCTAACGCTCTCACCAAC

GAGCAGAAGGAGAAGCTGAAGAAGCACGCGACCGAATGCCTCTCGGAGTCGAAAGTCGAC

ATGGCCCTGGTCCAGAAGATGAAGGGTGGCGATTACAAGACTGAGAATGACGCTCTGAAG

AAGTACGTGCTCTGCATGCTCATCAAATCGGAGCTGATGACCAAGGACGGCAAGTTCAAG

AAGGACGTCGCCCTCGCGAAGGTTCCCAACGAGGAGGACAAGGCCGGAGTCGAGAAGATC

ATCGACGCCTGCCTCACCAACAAGGGCAACACCCCCGAGCAGAGCGCATGGAACTACGTC

AAATGCTACCACGAGAAGAACCCAAAACACCCTCTGCTGTAA

> EsigOBP7

ATGTGGTTGGCTGCCTTAGCTCAAGATACATCGTTCGATAACATCAATGTGGAGGAATAC

AAGGATAGATTACGTTTAAGAGCTCTCATAGATTGTTTAAAAAGAAGAGGAGAGTGCACA

TGGGCGGAGGCTAATGTTAGAGACAGGGGCCAAGAAATAGTGGCGACTAAATGCCAGTCA

TGCTCAGACAGGGAGAAGCAAGTGATCCACGATCTGACCTCGCTCATCACCCAGGAGTAC

CCCGATGAGTTGGCCACGTTATTCCAGGCATACGATCCGACGGCCGAGAATACCAACAGT

TACCTGAAAGCGGTCAACACATTTTCTTCTCAAGGCAATATTCCTCATATTCCAAGGGAA

ACTATCGACCGGTTTTAA

> EsigOBP8

ATGGTCAGCATCACTACGAGATTTATTTTTACGTGTATCGTCGTTGGGGTACTACACTGC

AATGCTGCAATTCCTGACATATGCGCACATCCTCCTATGGTGAATCCACACGATTGCTGC

AAAGCGCCGAGTATTTTCGAGAAGGCTGTTATGGACGAATGTGGAGTTGAGCCGATGGCC

AAAGAAGATCGACCTCCTCACGAGAGACCACCGCCGTGCGATAAGCTTCTATGCCTATTC

AAGAAAGAAAAGCTCATGCTGGACGACGAGAAGCTAGATCTACCAGCAGTGAAAACTTAT

ATTAGCAAATGGGTGGAGAAAAACCCAGAGTGGGCTTTTATTGCGGCCGACGCTAACGAG

CATTGTTACTCCGAGCACATGCTTCAGCATGGACCACCAGATCTCTGCCAGCCTTTCAAG

ATTTCGTTCTGCCTTGCAGGAGCATTCTTCTCTTCCTGTCCTGCTAAACATTGGAAGGAA

TCCATTGCCTGTGGATCATTGAAAAAGTTCATGGACGAATGTCACATTCAAGAACGTCCT

GACCAGTAA

> EsigGOBP7

ATGTTTTGTCTGGTTTTCGTGGCTGGTTTGAATTTGGTGCTTTCGAAGCCAGATGGACTT

ATCATGAAAACTATCAGCGTTGGCTTCGGAAAAGCTTTGGATGAATGTCGTGAGGAGAGT

AATCTAACACCGGAAATAATGGAAGAGTTCCTCCACTTCTGGAGGGACGACTTTGAGATC

AAGCACCGGGAGCTGGGTTGCGCCATCCTCTGCATGAACAAGAAGTTCGATCTCCTGCAG

GACAACCAGAGGATGCATCACGGGAACATGGACGATTTCATCAAATCCTTCCACCAGGGC

GAGAAGTTGGCCCGTCGCATCATCGAAATCATCCACGAGTGCGAGAAGGAGTTCGACGCC

ATGGAAGATCATTGCGATAGGATAATGCACGTCGCCAAGTGCTTCAAGAGCAACGCGATA

AAGGAGGGCATTGCGCCGACCATGGAGATGTTGGTCATCGCCGTCTGA

>EsigGR1

ATGGTTAACCAGAAGAGCAACGTTATTCTCGCGGTGTTCGCTTTCGCGTGCTGCGCTGTT

TTTTGTGATGCATATCCGTCGCACTACCAATTACTACCACCACGGCCGGGCTACATCCCC

GTTTACATCCAAAACGGCGATACGCCACTCGAAGAGATAAACCTGGAATTAGCCGAGGCT

TTCCACGCCATCCCGTCGGGTCGCAGCGCGAACAAACAACTCTTGGCAGCAGAAAACGAA

GCCGTCATTCATTACGACCCCATCAAGTCGGAAAAAACGAACGTGATTACGCTGGAGGAC

GCGGCAGACGGTAAGATAAATTTGGTGCCAATCGAGAGCGAAACGCCGGTCGAGCCAGAG

GAAAAAGCAACGGATATAGAGATTATTATCAACAAGCAAGAGGAACCCGAATCCAAGTAC

ATTCAGAAAATTCCGCCTTCGCAATTTGAACAGTCTTCAGCGTACAATAAAAATGTTTAA

>EsigOR1

ATTATTATTTGCGTGACCTTGTACCAGTGCGCGACAAGATCCGGTATGAATCTTGACTTC

GTGTATATGATTCAGTACCTGTTAACAATTACATTGGAACTCTTCTTCTACTGCTGGTTT

GGAAACGAGCTAACTATAGAGAGTCAACGCCTATCGCAAGCGGTGTTCGACAGCGAGTGG

ATGGGGGCACCACGGGAATACACCTCTATTTTGAGGATCATTCACGCGAAGAGTTTGAAG

CAGACCACACTTACAGCACTCGGCGTGTTTAAGCTG

>EsigOR2

CCGTATTTGTACCTGATTTTCACACTGTCATCCGCCGAAATTTGCGTACTCATGTTCCAA

TTCACCGAGGAACTGTTTGCCGGTCGCTTCCAGTACGAATTTCTTGCGTTGTTAACTTGT

TCAACACTCTTAGTGCATACCATGTCGTCGTATGGAGACAAAGTTATGAGACAGTGCGAG

TCGGTGGGCTCTGCTGCGTATCGCAGCGGCTGGGAGGGTTGGCCGGACATGGCTGCGCGG

CGTGACCTGCTCCTTATTATGATGCGTACCGCCAGACCTACCACTCTCAGGACCATTTTC

AGCGACATCAATCTCGCCGCTTTTAGCGCTACTGTCAACTCCGCATGGTCCTACTTCACA

CTTCTACGAACCAGCAGCGAATAA

>EsigSNMP1

CTCCAAATCTACAGCAGCCACGAGTTCGGGTCCCCCATCGTTGCTACATTTCCTCACATG

TATCTTACCGATGAAGTTTACTCGAATTCAGTAGAAGGAATGTTGCCAGATCAAGAAAAA

CACGAGGTATTCGTTGAGATTGACCCGTTGTCTGGAACAGTTCTTAGAGGAGGAAAAAGG

ATTCAGTTCAATATGATTCTTAGGCCTGTTCCATTTATTACAATGACAAATAATCTAGAT

GTAAGTTTAACACCAATGCTGTGGGTTGAAGAGGGTATAGAGCTGCCACAAGAATATGTG

GACGAGTTGCAATCGAGTCTCTTCAATCAGTTGTTCTTGGTAGAGGTTTTGACATGGGTA

TTTTTAGTGGCAGGTGTAGTTATCGCTTGTGGACTGCTGATTCTGGCAATATGTTGCAGT

CGTAGCCATACACGGTCCCTCAAAACTGTATGA

>EsigIR1

CGGGGCTACCGGCTGCTCGTCGAGCAGCACAGCTCGTCGCTGGCCATGCTCCAGAATGGC

ACCGGAGTGTACGGGCGGTTGGCGAGCCTGATGCGGCGGCAGGACACGTGGCTGGTGGGC

AGCGTGGAGCAGGGCGTGCGGCTCGTGCTGCGGCGCCGCGCGCACGCCGTGCTCGGCGGC

CGCGAAACTCTCTATTTCGACACTAAACGATTTGGCGCAAACAACTTTCACCTGAGTGAG

AAATTGTACACGCGGTACTCGGCGATTGCGCTTCAGATCGGATGCCCTTTCATTGAGAAC

TTCAACCTGGTCCTTATGCAACTGTTCGAAGCGGGCATCCTGGGCAAG

>EsigIR75p-1

ATGGGTGTATCTGTCTGTGGCATCATAGCTCCCAAACGGATGGACCGATTTTGATTTAGT

TATTTTTGTTTAAAAGGTGACTTGGTCGAAAGTGTTCTTAGCTATAATTCAAGAAAAGCC

GTTTGA

>EsigIR25a

TTCCACAGGTCATTGCTGGACGCTGGCAATTGGCCAAAAGACATGAAGTATCTGAGCTGC

GACGACTACGACGGCACGAACACACCCAACCGATCGCTCGATCTCAAGTCGGCCTTCGAT

CAGGTAAAAGAAGCACCAACCTACGCCCCTTTCTACTTCCCCGACGATGATGTGGTGAAT

GGTAAGAGCTTCATGGAATTTAATACTGAGATGTCGGCTGTTACCGTCAAGGACGGTGCG

TCTGTGGGTAGTAAGTCGTTGGGTACCTGGAAAGCTGGCCTCAGCAATGAACTCAAATTA

AATGACCCCACCATCATGAAGAACTACACAGCCGAGATTGTTTACAGAGTTGTTACTGTT

GAGCAAAAACCATTCATGATGAGAGACGACAGTGCACCTAGGGGTTTTAAAGGATATTGC

ATAGATCTCATAGAGGAAATTCGACAAATTGTCAAATTTGATTACGAAATAATGGTAGCT

CCTGATGGAAATTTTGGAAATATGGATGAGAAGGGCAACTGGAACGGTATTGTTAAAGAG

TTAATGGAGAAACGAGCCGACATAGGTCTGGGTTCAATGTCTGTTATGGCTGAGAGAGAG

AACGTCATTGACTTCACAGTGCCTTACTATGACCTGGTCGGTATCACAATACTGATGACT

CAACCAAGATCTCCTACTTCTCTTTTCAAGTTTTTGACAGTTCTCGAGAATGACGTGTGG

TTATCAATATTGGCTGCTTATTTCTTTACCAGCTTTTTAATGTGGGTCTTTGACAAATGG

AGCCCGTACAGCTTCCAGAATAACAGAGAGAAATATAAGGATGATGAAGAGCAGCGAGAG

TTC

>EsigIR2

GATGATGAAGAGCAGCGAGAGTTCACATTAAAAGAATGCCTGTGGTTCTGCATGACATCG

TTAACCCCCCAGGGTGGAGGGGAGGCTCCTAAGAACTTATCGGGGCGACTATTAGCTGCC

ACTTGGTGGCTGTTTGGATTCATAATAATAGCATCTTATACTGCTAACTTGGCCGCTTTC

CTCACCGTGTCACGATTGGATACACCAATTGAATCACTGGATGATCTATCGAAACAATAC

AAGATACAGTACGCTCCACTCAATGGATCATCAGCCATGACTTATTTTGAGCGAATGGCT

AATATTGAGGGCAGGTTTTATGAAATATGGAAAGAAATGAGTCTGAATGACAGCCTGAGT

GAGGTGGAACGAGCCAAACTTGCCGTATGGGACTACCCCGTCAGCGACAAGTACAGCAAA

ATGTGGCAGGCCATGAAAGAGGCTGGTCTGCCCAATTCTGTCGAGGAGGCCGTTCAAAGG

GTTCGCGATTCCAAGAGTTCCAACGAAGGCTTTGCTTGGCTGGGAGACGCGACTGACGTG

AGGTATAACGTGCTCACCAGCTGCGATTTGCAGATGGTGGGTGATGAGTTCTCGAGGACA

CCGTATGCGATTGCTGTGCAACAAGGCTCGCCTCTGAAGGACCAGTTTAACAATGCTATT

CTCCAGTTGCTCAACAAACGGAAACTCGAGAAACTCAAAGAGAGCTGGTGGAACAGCAAC

AAGGAAGCAAAGAAGTGTGAGAAGCAGGACGACCAGTCGGACGGCATTTCTATTCAGAAC

ATAGGAGGTGTCTTCATCGTCATCTTCATGGGCATTGGTCTCGCGTGCATCACTCTAGGT

GTGGAGTATTGGTGGTACAAATGGAGAAAGAAGAGTAAAGTCCTTGACATCAGACAAGTG

GATCCGATCACTATGAAACCGCATCACCACAATAAAGTCGACTCGAGGAATGAAGATAAC

CTGCCTAATAATTCTGAGATTATGTCTGGCTTCAGATCAAGAAATTTTGGCATATCCACA

TTGAAACCACGATTTTAG

>EsigIR3

TGGATTGCCAGGGAAGGAAGCGCTGTGGAATATGTCATCGCCAACCCCAATGAAGACTTA

TATTTCCTGAACAAAATGGTCACGTCCGGTCGCGGTCGGTACAGCAGGCTAACGGAGGAC

AAAAGCTATCTAAAGGAATTAACCCGCAACGTGATCTACATTCGCGACAGGCCAAGCGTC

AACCACCTAATCTACAACGACTACCGGGAGAAAGCACGGAGCGGTGTCGAGGAAAAATCG

AGATGTACATACGTCATCACCGCCAACCCCTTCATGATACGATCTCGGGCTTTCGCTTAT

CCAATTAACAGTACTTTACCGGCACTGGTCGATCCAATACTAAGATCGTTTGTGGAGGGT

GGCATCGTTGAATACCTCGAAGAGCTAGATCTTCCCGCAAATGAGATTTGTCCACTCGAT

TTGCAATCAAAAGAAAGGCGTCTCCGGAACACCGACCTCACCATGACGTACACATTGGCT

GGCATTGGTCTCTGCGCAGCAATCGCTGTATTTGTAGGAGAGTTGCTGCATCGATATTGG

AAGGCGCGACGCGGTGACTACGATGATGAAATCGACAATCAGCTGTTTAAAAAGAACAAA

CAAACCAAGTCTAAAATATTTTCGGAGCGTACTCGACCTCCGCCGTACGAATCGTTGTTC

GGATCTAAAGACAAGAGTTCATTTGAAAACAGCCAGAAACGCATCATCAACGGCCGCGAC

TACTGGGTCATAAAGAATTCCCTGGGCGAGGTGCGACTCGTGCCAGTTCGAAGTCCCTCC

GCGTTTCTTTTTCAATAA

>EsigIR40a-1

CCTTGGCACTTCGTCAAGATCAACAACGACTCAACTGTAACAGTGACGGGAGGCAGGGAC

GAGAAGCTGCTTGCTCTCTTGGCAGAGAAACTCAATTTCAGATATGAGTATTACGATCCT

CCGGACCGCAGCCAAGGCACTGGTATCACGGCCAACGGCACATTTGAAGGAATTCTTGGA

CTGGTGTGGAAACGGCAGGCCGACTTCTTCCTAGGCGACGTGACCATGAGCTGGGAGCGT

CTGCAAGGCGTGGAGTTCTCCTTCCTCACGCTGGCTGACAGCGGCGCCTTCGTCACTCAT

GCGCCCTCTAAACTGAGCGAAGCGCTAGCGCTAGTCAGACCCTTTCGATGGGATGTATGG

CCTCTAGTGGTGTGCACCATGGTGCTAGCAGGGCCAGCGCTTTGGGCCGTAGTGGCGGCG

CCAGCACTCTGGCAACCGGCGCTGCGACGCCGCAACCAACTCCAACTCCTCGCCAGGTGC

TGCTGGTTCACCACTACGCTTTTCCTTAGACAATCGGCGGGCCGCGAGCCGGCGCACACG

CACAAGGCGCGCCTGGTGTCCGTTTTGCTGTCACTGGCAGCCACCTACGTCATCGGCGAC

ATGTACTCCGCCAACCTGACC

> EsigIR75p-2

ATGTATCCTATTTTGGAAAACGAGGCTCAAGGTTCCGGTATTGAAAACGTAGTGAAAAAA

GAAAGGACGAGGCATAAAAATACGAGGAAAAAGAACTCGCTCCAGTCGGGCCAGAATGGT

CAGTGCGAGACCGTCGTCGGCGAGAGGAGAAAGTTCTTAGTTACAAAGCTACCGGCGGAC

GAAGCGGATGTCTCGCCGGTCCGCGCGAGGAACTACAACAATGCGCACACCATACACTTC

GGGAGCTCCACCGCGGAGCCACAGCGGCCCAGCGTCCGCGACATCTTCGACGGGAAGACG

GGCGACCTTGTCAAGTCCAACAGTTTCAAGGTGACAGCGGCTAAGGACGATAGTCAGTAC

AAATACCAGACTATTCACGGCACTTCGGTCAGCCAGCAGCGACCTTCCATATTCGACTTA

TTCCGGCCCAAGCGGAAGAGCGACGGTAAGAGGCCGTCTCTCATTCAGAACATCAAGTCG

ACGTTCTCAAAAAACTCCAATCAGCCGAGCAAATCTTCCGAAACCAAGTCGGAGACGCCG

CCGAAAACGGACAACGAGTCAGCGTCGAGCGATAAGAGATACTATCACACGGTGACTGGT

GCCTCCTCGCGCCGCTCTAGTCCCATCACAAAAGTGATGGATATATTTAGAAGTAAACCA

TCTCATGACACGAATCTTGAATTAGATAAGAAGAAAGGTCACGCGGCGGCAAGCCAAGCG

ATCTCGAGCAAACGGAGGTCATCCCTCGGCGCCGGCACTACCTATTACAATAAAGGCTCG

TACGCCTCGCTGGACCCGCTGCAAGCGCGTCAAGCCTTCCGCGAGGTGCGAGGACTGCCG

AAGTACGATCCTTATTTGTCCATTGTACAAATATCCATAGGCGGTAGAGATAAGTCACGG

CTTCTCATAAATTTCTTCAAGTATCACAAGTGTTATGAAATTTTGCCAAAATCCGCTAAA

GTCATCGTTTTTGATACTCAATTCTTAGTTAGGAAAGCTTTTACGACGCTTATTTCGAAC

GGCATTCGCTCCGCACCCCTTTGGGACTCAGATAAAAAAAGTTTAGTAGGCATGATTACG

GTCACAGACTTCATAATGATATTACAAATCCTTTACGAGAATCAGAAATCCATGGACGAG

TTGGAGGAGCACACTCTCAGCACGTGGAGAGTTCTTTTAAAGGGGCACACCAAGCAGCTT

TGTTTTGTTAGTCCGAATGCTTCCTTGCTAGATGCTATTAATATTTTAATAAATAATCAT

ATTCACAGAATACCCATTATAGATGAAGTTACCGGCGACGTACTTCACATTTTGTCACAG

AAAAGAGTTTTGCGCTTTCTATTTGTATATTTGAGCGAATTTCCTGAGATACAGTTTATC

CGTTCTAAGCTTCAAGATTTAAATATTGGTACTTACGATAATATAGAAACTGTTAGTGAA

TCTACGTCTGTATTGGAAGCTTTTGAGAAGTTTAACGCTAAGCGCGTGTCGGCTCTGCCG

CTAGTTGACGAGAACGGCGTTTTGATCGATGTTTACGCTAAGCACGACGTCATTAATTTA

GTAGCTGAGAAAACTTACAATAACTTAAAAATATCGCTGAAGGAAGCTTGCAGCAGAAAG

AAAGTGTGGATGGAGAAGTTGCAGAAATGTAATAAAAACATAACTCTCTACGAAGCACTC

GAAATAATTGTTAGATCGGATAGTCATAGGATATTTATTGTCAACGAGGATAATACTTTA

TTTGGTATCGTAACATTGACCGATTTATTGAAGCATTTAATTTCATCAACACCAAACGCG

AACAGGAACGTTCAGTTAGATCAATTATTTGCTCAGCCCAGTGGCGAAATTCAACACGAA

ATAACAGAAAGTCCAACCCACGAAGCAAAGTCGGATGACTCTGCAGATCAGCAAGCTGTT

GTTGAAAATTTGGAAACCGATAATAAAGAGGCGCAAGATCTCATAGATAATTCAATTAAT

AGTAATGAAAGCGAAGTAGATGCGGGGGTTGAAGTAGAGGCTCCCAATAATTCAGTAAAT

GAAAGCGAGAACGCGGAAATAATGACAAACGCCTAA

> EsigIR4

GAGCGCTTCTACCGGCACCTGCGCGCCGTGAGCGTGGAGGGCGAGGCGGGCCGGCCGCGC

GTGCAGTTCACGGCCGAGGGCGAGCTGCGCGCGGCCGACCTGCGCGTCGTCAACCTGCGG

CCCGGTGTCGGCGAGCAATTGCGCTGGGAGGAAATCGGCGTTTGGCAGTCGTGGCGGCGC

GAGCAGCTCGACATCAAGGACATCGTGTGGCCGGGCGGCAGCCACACGCCGCCGCAGGGC

GTACCTGAGAAGTTCCACCTCAAGATCACCTTCCTCGAGGAGCCGCCTTACATCCAGCTC

GCGCCGCCAGACCCCGTCAGCGGCCGCTGCCCGCTCGATCGCGGCGTCTTTTGTCGCGTG

CCCTCTCATGCGCCGGACCTCGGGGTGGAGGCAAATAACAGCTCGCTGCATCAGTGCTGC

TCAGGGTTCTGCGTGGATCTGCTTGAGAAGCTGGCTGAGGAGCTGGGTTTCACGTACGAG

CTGAGTCGCGTGGCGGACGGCCGCTGGGGTACCATGCAGCGTGGACGCTGGAATGGCCTC

GTCGCCGATCTTGTCAACAGAAAGACCGATATGGTATTGACTTCGCTGATGATCAACTCG

GAGCGAGAGGCGGCGGTAGATTTCAGCGTGCCATTTATGGAGACGGGCATAGCGGTGGTG

GTGGCGAAGCGCACGGGCATAATTTCGCCAACGGCCTTCTTGGAGCCCTTCGACACGGCT

TCGTGGATGTTGGTGGGTGCAGTCGCCATCCAGGCGGCCACCTTCACCATCTTCCTATTC

GAGTGGCTCTCGCCCAGCGGGTTCGACATGCGCGTGCGCGGCGCTGCGCCGCATCGCTTC

TCGCTCTGCCGCACGTACTGGATCGTATGGGCCGTGCTCTTCCAGGCGGCCGTCCACGTG

GACTCGCCGCGCGGTTTCACGGCGCGCTTCATGACTAATATGTGGGCCATGTTCGCCGTC

GTCTTCCTCGCCATTTACACGGCCAACTTGGCCGCCTTCATGATCACGCGCGAGGAGTAC

CACGAGCTGTCGGGCCTAGACGACGCACGGCTCGCGCGCCCGCTCTCCATCCGCCCACCG

CTGCGCTTTGGCACCGTGCCCTGCTCTCACACCGATGCCACGGTCGCCAAGTACTTTCCT

GAGATGCACGCCTACATGTCTAGTTACAACCGGAGCACAGTGGGCGCTGGCGTAAGCGGC

GTGCTGTCAGGAGAGCTGGACGCGTTCGTGTACGACGGCACGGTGCTGGACTACCTCGGC

TCGCAGGACGAGGACTGCCGGCTGCTGACGGTGGGCGCGTGGTACGCGCGCACCGGCTAC

GCGCTCGCATTCGCGCGCAACTCTAAGTACGTGCCCATGTTCAACCGGCGCCTGCTCGAG

TTCCGCGAGAACGGTGACCTCGAGCGGCTCAGGAGGTACTGGATGACGGGTACGTGCAAG

CCAAATAAGCAGCAGCACAAGTCGTCGGATCCGCTGGCGCTGGAGCAGTTCTTGTCGGCC

TTCCTGCTACTGATGGCGGGCATCCTGTTGGCGGCGCTGCTGTTACTGCTCGAGCATCTT

TACTGTCGTTATGCGCGCGCCCCGCTCGCCGCTAGTCGCGCGGGATCCTGCTGTGCGCTC

GTCTCGCTCAGTATGGGTCAGTCGCTGACGTTCCGCGGGGCCGTGCTGCGAGCGGCGGCG

GCGCGTGCGCGCGGCCGCTGCCGCTCAGCGGAGTGCGGCGCGATACTGTGGCGCACGCGG

> EsigIR76b

GCTGCCGCCTTCCTCCCAATTCTAACCGAAGCCCGATCGATAGTCCGATATTCTGTGGCG

CTGGATGAGGGCATTTGGATGATGATGCTAAAACGACCGCGCGAGTCTGCCGCCGGATCC

GGTCTGCTAGCACCTTTTGACGAGAATGTCTGGTACCTCATCCTAGTAGCGGTGATAGCT

TACGGGCCCTGCATCGCTATCATCACG

>EsigIR93a-1

AGGAAGGACACGTATTTGGAGATGCAGTTGAAGGAATCTCTGGAACCAAAATACAAAGCA

CTGTTAGCTGGCGCACGGCTCCTGCCCCGAGGGCACCTGCCCATGGAGGGCGCGGGCGAT

GCGGAGACGGAACGCGTGCGTCGCGGCGGCCACGTGCTGCTCGACTGGAAGATGCGACTC

CACTACCTGATGCGGCGCGATCATCTGGAGACTGACACCTGTGACTTCGTACTCAGCTTG

GATGAATTCATGGATGAGCAGATGGCGATGATAATGCCTGCAAACAGCCCGTATCTTCCA

ATAATTAATGAAGAAATAAAGCGCATGCACCAGATGGGCCTGATCCACAAGTGGCTGTCG

CAGTACCTGCCGCGGCGCGACCGCTGCTGGAAGAGCTCA

>EsigIR5

ACTTACACGCTATTGTATGACGATGATTATGGTCTAATCCGGCTGGAAGCGGTGCTGAGG

CTGCCGCGTCGCAACACGGTGCGCGTGCGCCGTCTCTCGGCCGACAATGCGGAGCTCTTC

AAGCTGATGCACCACAACAAGGAGTACAACGTGCTGCTAGACTGCGCGGCCGACCGCGTG

CTCGGCTACCTCGAGCAGGCCATGCAGGTCAACATGTTCTCCGAGTATCAGAGTTACATC

TTGACTTCTCTGGACGCTCACAGGCTGGATTGGGACAGCATGGTGGGGGGTCGCAGCAAC

GTCTCCTGCCTCCGCATGATCGACCCCGATGACGACGAGATGAATATACAGACCAGAAAT

CCGCCCAATCGTTACGCTACCCTGGAAGGAATATTAGCAGCAGATGCAGTATCTGTGCTG

GCAGTGGCACTGGAGACATTCCAAGAAGGGCACTCTCTAGAGACATTCGGCGCTGAAAGT

TGTGATAGGGACGGGGGTTGGAAGCACGGCTTGGATTTGGCAGAGCACATGAGACAAAAT

CACGTCCAAGGCTACACGAACAGTATAGAGTTCGATAGCAATGGTCAGCGTGCCAACTTC

ACTTTACAAGTGCTGGAGAGAGAACCGAGTGGCTTTATGGTGATAGCCGAGTGGGAGGCA

CAGACAGGCGCCGTTGTGCAGGGCGGCGATGCTGACAGCAGAGAGGACACTGTCTTGGAA

CGAGCCCATGACAAGGTTTTTACGGTAGTATCACGCAAGGGCCTGCCCTATTTGGACGTT

ATTAAAGATGACACCTTAAAAGGTAATGATAGGTACCGTGGCTACGCTGTCGATCTCATC

GATGCCATTGCAAAGATCCTAAACATCAAATATGAATTTAAAGTACTGGAGGGAGGATAT

GGCACACGAGACAAGACGACTAATAAGTGGAACGGTCTCATCGGACAACTTGTTGATGGG

AAAGCAGATATGGCTATATGTGACCTTACCATTACTTATGAACGCCGAAGTGCAGTAGAT

TTCACAATGCCATTCATGACGTTGGGTATCAGCATTCTATATAAAAGAGCCGAAAAGCAG

CCACCCAGCATGTTTTCCTTCATGGCACCTTTTTCAAATGAGGTGTGGCTTTATGTTGCA

ACGGCGAAGTTGATTGTATCTATGTTGCTGTATTTATGCTCAAGATTATCGCCAGGCGAC

TGGGAAAATCCTCATCCATGTGACAAGGAACCTGAGGAACTGGAGAACATATGGAATTTG

AAGAACTGTGCCTGGCTGACTTTGGGGTCCATTATGACACAGGGTTGTGATATCCTACCC

AAAGCATTTGGTACACGTTGGATAACTGGCATGTGGTGGTTTTTCGCATTGATCCTGACT

TCATCTTACACAGCTAACCTAGCGGCTTTCCTCACTAATGATCGTATGGAAAAAACAATT

CAGGATGTTAAGGATTTGAGTAACCAGAATAAAATTAAATATGGGGTCCTGGAGGGTGGC

TCTTCTTATAACTTTTTTAAGGATTCAAATGATTCCATATACCAGCGTGTTTGGACTACA

ATGGAATCAACAAGACCTTCCGTTTTTGTGTCCGACAATAAGTATGGTGTGGAAAGAGTT

CTACAAAGAAAGGGAAAATATGCCTATTTCATGGAGTCGACGAGCATTGAATACATCATG

CAGCGGAAATGTGATCTTTATCAGGTCGGCGGTAACTTAGACTTCAAAGGATATGGTATA

GCTTTACCGATGAATTCACCATATCGGAAGAATTTCAACAAAGCCATTCTTAAACTGCAG

GAGACGGGTGAATTGGACAAGCTAAAAAAGAAATGGTGGGAGGAAATGGATATAGAGAAG

AAATGTGATGAGGAATCTTCGGGCGACTCAGAGGACAGTATGGAATTTGGCTTGGACAAT

GTGGGTGGTGTATTCCTTGTGGTGGCCATGGGCTGTGTGCTGGGTGCCATGGTCACAGGA

ATCGAGTTCCTATGGCATGCTCGCTCAATGGCAGTAGATGAAAAGATAAGTACAAAGGAA

GCAATAGTCAGTGAGCTAAAAGCGTCTTTGAACTTCAATGAACCAACAAAACCTGTATTG

AAGGGAAGATCGTCGACTAAATCTCCACCACCCACATCAGAAATGTCAGGAAAAACATTT

TAAATGCGCGCCGTTTCTTGA

> EsigIR6

ATGGAAGCGACTATCTCTGATCCGACTAACATTGGTGCAATATTCCAAGGAGAAGAGCGG

TGGCTGGAGGCGTCGTTCCTGGTGGCGGTCGACACGGTGAACGAGCGGCGATCGGGCGCA

GAGCGCGAGCTGGCCGCGCTGGTGAGGCGCGTGCCGGCCGAGAGCGCGCTCGACGCCGAG

ATCGCGGCTTGCGATTTGTTTAAGGAGAAGGTAGCGGCTGTGTGGGGTCCGGCTTCGCCC

GCGTCCGCGTCGCACGTGCAGTCGGTGTGCGACGCAGTGGAGATCCCGCACCTCGTCAGC

TTCATGGACGAGCACCAGGCCAGGAACTGGTCCGTCGTCAACCTACATCCGCACCCCACC

GTCTACGCTGACGTTCTCTTTGACCTTGTTTCCTCGAAAGGCTGGCTGAAATTCACGATA

ATCTACGAGACTGTCGAAGGACTGGCGAGGATCAAGAAGCTGTACGATTTGGCGCAGGAA

GATATGGCATACACGATTTCGATTTATCAACTGTTGGCCACGTCTGAAAATAACTACAGA

CCAATTCTTCACAAGATAAAAAAGTCGGGCGACACAAATTACATACTAGACTGTCCGGCA

GATTTGCTCGACACCATACTGAAGCAAGCGCAACAAGTCGGCATTATGTCCGACCAGCAC

AGCTACATCATAGTTACGCCCGACATGCACACTATAGACTTGGAACCATACCAGCACGGC

GAAGCTAATATTTCAGGTATACGCTTTACAGATAGCCAAGATACGGAAGTATTACAATTC

ATCGATAAATTCAATGTAAAATATCAAGAAATAACGGAGACAGAGATTACCGAAGATGAA

AAATTGTCTGGCGACAGCATGATACTAGAAATCGCGCTGGTTTACGACGCTGTCCTGATG

TACGCCGAGGGTCTCAAGGATTTGGATGCGAAATTCGAAACTGAGAACCTGGATTGTGAG

AAGGGAGATAGTTGGTCTCACGGGTCTTCAATTATCAACATGCTTAGAGTTAAACATACC

AATGGATTGACAAGAAGAATAGGCTTTAACGATGAAGGTTTACGTACAGACGTACACGTT

GATATAGAGGAACTAACTATGGAGAACGGTCTGGAGAAGGTGGGATCATGGAATCGATCA

AGAGGATTAATATTTGAACGACAGCCTATCCCACCGAGCAATATAACAGTAGATGATACG

TTAGAAAATAAAACTTTTATCGTGTTAACCGCTCTGAAAGCACCCTATGGAATGTTGAAA

GACTCTGCAATTAAACTAGAGGGCAACGATCGCTTCGAAGGTTTCTGCATTGAGCTGATA

GAGGAACTCTCCAAGATGCTCGGATTCAATTACACCTTCGAGATTCAGAAAGATCGCATC

TCTGGATCATTGGACAAGAAGACCAATACCTGGAACGGCATGATCAAAAGGATCATGGAT

GGCGAGGCTGACTTTGCTATAACTGATCTGACAATAACGGCAGATAGAGAGAGCGCCGTG

GATTTCACAAGCCCCTTTATGAACTTGGGAATTAGCATTCTTTTTAAGAAACCGTATAAG

CTGCCCCCGAGCCTCTTTTCCTTCGTCTCGCCATTTTCCCAAGAAGTTTGGTTTTACTTG

GTCGGTGTCTATATCGGAGTATCTACACTGCTATTCGTCCTAGGCAGGATATGTCCCGAC

GAGTGGCAGAATCCGTACCCGTGCATTGAGGAGCCAGAATATCTCGAGAATCAATTTTCA

ATGGCCAATTCGTTCTGGTTTACACTGGGAAGTATTCTAACACAGGGCTCTGAAATAGCG

CCAATAGCACTTGGGACCAGAATAGTGGGGAGTATTTGGTTTTTCTTTGCTTTGATCATG

GTGTCGTCCTACACAGCCAACCTGGCGGCCTTCCTTACCGTCGAGAATAAAGTGTCTGAA

ATCAACAGCGCCGAGGAACTGGCGAAGCAAACTGCTATTGCATATGGAGCCCAAAAGGGT

GGATCGACGCTCAATTTTTTTAAAGAATCTGACAATCCCATTTATCAAGAAATGTACAAT

TACATGTCAAGTCATCCCGATGTTCTAATGTCTACTAATGAGGAAGCAGTCGAGAGGATA

AAGAATGACAACTACGCATTCCTGATGGAGTCGACGTCGATAGAATACACGGTGGAACGA

GAGTGCGAGGTGACGCAAGTGGGAGGGCTTATCGATAACAAGGGATACGGAATCGCCATG

AAGAAAAACTCGCCGTATCGAAACAAGATGAGCGGCGCCGTCCTTCAGCTGCAAGAGCGG

GGCAAACTGACACGTATGAAGGACAAATGGTGGAAGGAGATGCGTGGTGGCGGAGCCTGT

GGGGGAGAAGAGGATTCAGGCGGTGGAGCCACGCCGCTGGCGCTAGCTAACGTGGGCGGT

GTCTTCATCGTCTTAGTCACGGGCTCATCACTAGCAGCCGTTTGTGCCTTCATCGAAATG

CTCATCAAGACTTGGGCCGTGTCCTTGAAATATAAGGTATCGTTCCGTACGGAACTAATC

GAGGAGCTAAAGTTCCTGGTGCTGGGCGCGGGCAACACGAAGCCGGTGCGCGCGCACACC

ACGCCGCCGTCCACGCGCAGCGCCTCGCCCGCCTCGCGCCGCACCGCCCACTCGCGCCAC

AGCATCCACTCTGCGCGCAGCCACTCCGTCAAACTGTCGCAAAAGAGAAGATACACGTAG

> EsigIR7

CCGGCCGAGCCCGCCGAGCCCGTGCGCCCCGCTCCCATCTCCTTCGCCCTCGTCGTACCC

CACAAGTCCTTCGGCCAGCGCGACTACGTCAAGGCGGCTAAGGCGGCCCTCCAGACCGTC

GAGCGCAAGTTGAACCTCGTCGGATTAACATTCAACCTCTCGATGCAGGAGCTCACGCCG

AGCCCTATGACAATCCTCGACTCGCTGTGCAAGGAATTCCTGGCGTCAAACGTTTCGGCC

ATCCTCTACCTGATGAACCACGAGCAGTACGGACGCAGCACGGCCTCGGCGCAGTACTTC

CTGCAGCTCGCCGGCTACCTCGGCGTTCCGGTCGTGGCTTGGAACGCCGACAACTCGGGC

CTCGAGCGACGCGCCTCCGCGCTGCGTCTGCAGCTCGCGCCCTCCGTGGAGCACCAGGCC

GCTGCCATGCTCGCCATCCTCGAGCGCTACAAGTGGCACCAGTTCTCGGTCGTCACGTCC

CACATCGCCGGACACGCCGACTTCATCCAGGCTGTGCGCGAGCGCGTCTCCGCCGTACAG

GAGCGGTTTAAGTTCACGATCCTGAATGCGGTGCTGGTGAGCGCGGGTGGCGCCGAGCTG

AGCGAGTTGGTGGCGAGCGAGGCGCGCGTCATGCTGCTCTACTGCACGCGCGAGGAGGCG

GCCGACATCTTCGCGGCGGCGCGCTCCATGCAGCTCGCCGGCGAGAGCTACGTCTGGGTC

GTGACGCAGTCCGTCGTCGGGCCCAACCGCCACGCGCACCACAATCTGCCCGTCGGCGCG

CTCGGCGTTCACTTCGACACGTCGCACGCGTCACTCATAAACGAGATCGCGACGGCGATA

AAGGTGTTCGCCTACGGCGTCGCGTCTTACAGCGCGGCGCCGGAGAGCGTGGCGCACCCG

CTGGGCGCGCAGCTGTCGTGC

> EsigIR8

ATGGAAAGCGGACCCTTCATTTTTATTCTAATTCTACTGTACAATATTGAAAATGGTCAA

ACTCGAAGACCTCCTGTTCATTTGGGCGGCATCTTCGCGTACGACAACCTGGCGAAGGAG

GCCGCGTTCCGAGTGGCGGTGCTGGAGGCGGGCCGGCGCGAGCAGGTGGCGCTGCGGCCC

GCGGTGAAGCGGCTCGAGGGCGTGGAGGCGGACGAGGCGCTCACCGCCGCGGACGAGGCC

TGCGATCTCATCAAGCAAGGTGTAGTGGGCATACTAGGTTCGTACGAACGCCCGTACTCG

CAAGCGGTGCAGTCCGTTTGCGATCTGACGGAGGTGCCGCACATCACAACCAACATGGAC

GGCGAGCAGACCAGGAACTTGTCGCTCATCAACCTGCATCCCCATCCTGAGGCCTACTCC

TTGCTGCTGACAGCTCTAATCGAGAGTAAAGGGTGGTCAGAGTACACAATAATGTACGAG

GACAGCGACGGCCTGGCTCGAGTGAGCGGTCTGATCCACCAACAAGGGCCAGACAATGTA

GTTAGCATCACGCAACTAGAACGAACTCCGGTTACCAATTACATACCCGTCCTCCAAAAC

GTTAAATCCTCCGGTGCAAAAAACTACGTACTGGATTGCAGAATAGAGATCCTGGACGAG

ATCCTGCGACAGGCGCAGATGGTGGGCATCCTATCTAATGAGCACAGCTACGTCATAACC

ACACCGGACATGCACACCATCGACCTGGAACCGTACAAGTACGGAGGAGCCAATATTACC

GGCATCAGATTGATCAATTCGACAAAAGAAATAGTAGAAGAATTTCAGAAGAAGTTCGAG

ATGACCTACCTGAAGCTGCTATCGGAGAGCGGCGTGGAAGCGGACCGCAGCTTGGTGGAG

TCCCCCTTGAGGGTGGACATCGCCCTGGTATACGACGCCGTGCTGGTCTACATGGCCGGA

CTCAAGCAGATCTTCAACTTCAGGGAGAACTCTCAAGTGCTTAAATTGAATAGTCCAAGT

TGCGAGATGTCGAGGAGCTGGATTCACGGTTCGACTATCGTTAATTACGTGAGGCTGAGT

AACATCACGGGGCTCACCGGCGAAATCGTATTCAATCCAGAAGGCCGTCGCATGAACACG

TCGTTTGAAGTTGTCGAGTTGGGACCTGACGGCGTCGAAAAGATTGGCGATTGGGATGGT

GTCAAATTGACATTCGACAGAATAACGAGGGAGACCGAAGTAAATCCAGATTCGACATCG

CCAGTGAAGAATAAGACTTTCATCGTCATAACATCTTTAACAGAACCATACGGCATGCTG

AAGGAAAACTCCTTAAAATTGGACGGCAATGATCGTTTCGAAGGCTTCGGCATCGACCTG

ATAGACGAGATCGCGACCCTGCTCGGCTTCAACTACACCTTCATCATACAGGAGGACTCG

GTGTACGGCAGCCGCGACAAAGTGACCAACACGTGGAACGGTATGATCGGCAAGGTACTC

AACAAGGATGCCGACCTGGCCATTGTTGACTTGACAATAACATCGGACCGTGAGGACGCC

GTGGACTTCACCAGTGTCTTCATGAATACGGGCATTAGCATTCTGTATAAATCCGCAACG

AAAGCTACATCCGGACTCTTCTCGTTCATGGATCCCTTCTCGTTAGAGGTATGGCTATGC

GTAGGCGCCGCCTACGTCAGTGTGTCCCTCCTCATGTGCGTGCTGGGTCGGCTCAGCCCC

GGCGAGTGGCAGAACCCATACCCCTGCGTGGAGGAGCCTGAGGAACTCAGCAACCAGTTC

TCCGTTTCTAACTCTTTTTGGTTCACGCTTGGTAGTATCCTGCAGCAGGGTTCGGAAATA

GCGCCAATTGCTCCTTCGATCCGCCTGGTCGGTGCGGTCTGGTGGTTCTTCACCCTGGTA

ATGACCTCGTCCTATACGGCTAACCTGGCTGCCTTCCTCACATATGAAATCAACAGCTCA

CCCATCAACAACGCCGAGCAACTCGCCAATCAAGACACCATCGCTTACGGCGCCAAGATC

AACGGATCCACACTGTCTTTCTTTCAGGAATCAACCGACCCCGTATACAAAAAAATGTCT

GAGTACATTGCCAAAAATCCTCACATGCTGACGAAAACCAACAAAGAGGGAATCGATAGA

GTCAAAGAGTCTGATTACGCTTTCCTGATGGAGTCGACGTCAATAGAATATACGCAGGAG

AGAGAATGTGACGTTACACAAATAGGCGGATTACTTGATCACAAAGGCTATGGCATTGCC

ATGCGGAAAAATTCATCATACCGCAATGAACTAAGTGCGGCTGTTCTCAAGCTTCAGGAG

CGGGGAGTGCTGACAAATTTGAAGAACAAGTGGTGGAAGGAGAAGCGGGGTGGTGGCGCT

TGTCAGGAAGAGGACCAGGGCGACGCGCAACCACTCAACCTGGTCAACGTGGGCGGAGTG

TTCGTCGTACTCGTGTTCGGCTGCTTCGTCGGCATCATGATCTCCTTCATCGACATGGTC

GCGGCGACCTACCAGAGAGCTAAGAGGGACAAGACGGCGTTCCGCGTGGCGCTCATGGAG

GAGCTGCGGTTTGTGGGCAAGTTTAGCGGGCAGACGAAGCCGACGCGGCTGCCCTCGCTC

AGCGACTCCGACTCGAGCCGCTCGGTGCAGTCGGCCGACACGCGCCACTCGCGCCACACC

CTCCACTCCCACCGCAGCCACAGCGTCAAGATAGCCAAACAGCGACGATACTCTTAA
