# Supplementary material for: Olfactory Proteins and Their Expression Profiles in the Eucalyptus Pest Endoclita signifier Larvae
Source: Front Physiol. 2021 Jul 19;12:682537. doi: 10.3389/fphys.2021.682537 (PMC8327093; doi:10.3389/fphys.2021.682537)
Supplement: Supplementary file 2 [file Table_2.DOCX]

Olfactory proteins and their expression profiles in the Eucalyptus pest *Endoclita signifie*r larvae

Xiaoyu Zhang^2^, Xiuhao Yang^3^, Hongxuan Ma^4^, Xiumei Liu^4^, Zhende Yang^1^, Ping Hu^1, 2*^

**Supplementary file 2 The protein names and gene accession numbers were used in phylogenetic trees.**

| OBPs | |  | CSPs | |
| --- | --- | --- | --- | --- |
| GB No. | Name |  | GB No. | Name |
| ABY71034.1 | PxylGOBP1 |  | KY810184.1 | HarmCSP20 |
| ABY71035.2 | PxylGOBP2 |  | KY810185.1 | HarmCSP21 |
| ACI28451.1 | PxylPBP1 |  | KY810186.1 | HarmCSP22 |
| AEB54587.1 | HarmOBP6 |  | KY810187.1 | HarmCSP23 |
| AEB54584.1 | HarmOBP4 |  | KY810188.1 | HarmCSP24 |
| AEB54582.1 | HarmOBP3 |  | KY815026.1 | HarmCSP25 |
| AEB54581.1 | HarmOBP5 |  | KY815027.1 | HarmCSP26 |
| AEB54580.1 | HarmOBP1 |  | AGH20054.1 | HarmCSP16 |
| CAC08211.1 | HarmGOBP2 |  | AGH20053.1 | HarmCSP15 |
|  |  |  | AFR92096.1 | HarmCSP12 |
|  |  |  | AFR92094.1 | HarmCSP10 |
|  |  |  | AFR92092.1 | HarmCSP8 |
