# Supplementary material for: Olfactory Proteins and Their Expression Profiles in the Eucalyptus Pest Endoclita signifier Larvae
Source: Front Physiol. 2021 Jul 19;12:682537. doi: 10.3389/fphys.2021.682537 (PMC8327093; doi:10.3389/fphys.2021.682537)
Supplement: Supplementary file 3 [file Table_3.DOCX]

Olfactory proteins and their expression profiles in the Eucalyptus pest *Endoclita signifie*r larvae

Xiaoyu Zhang^2^, Xiuhao Yang^3^, Hongxuan Ma^4^, Xiumei Liu^4^, Zhende Yang^1^, Ping Hu^1, 2*^

**Supplementary file 3 Primers were designed for fluorescence quantitative real-time PCR**

| **Gene** | **Primer** |  | **Gene** | **Primer** |
| --- | --- | --- | --- | --- |
| EsigOBP1-F | TTGTGTCGTTCCAATGGCTA |  | EsigGOBP7-F | TTGGATGAATGTCGTGAGGA |
| EsigOBP1-R | AGCGAGAAAAATGCCGACT |  | EsigGOBP7-R | CGTGGATGATTTCGATGATG |
| EsigOBP2-F | GGACGAGGAGATCACCCAAC |  | EsigCSP1-F | TACGATGGCATCAATTTGGA |
| EsigOBP2-R | AGCGCTCAGTTCTTCAGCAT |  | EsigCSP1-R | GAGGTGTCCGATGACCTTGT |
| EsigOBP3-F | TCGATTCTCGAGGAATGCGG |  | EsigCSP2-F | GCGAGCTAAAGAACCACCTG |
| EsigOBP3-R | AAGTCACTTTGTTCCGCCCA |  | EsigCSP2-R | GAAGGCCTCGTACTTGTTGC |
| EsigOBP5-F | TTACTGGCTTGTGCTTGCAG |  | EsigCSP3-F | TGTTTATCTGCTGCGTCGTC |
| EsigOBP5-R | GCCAAGCTTCTTTGCATTCT |  | EsigCSP3-R | GCACTGAGGACACGAACCTC |
| EsigGOBP1-F | GAAGATCGAGCGGGTGATTA |  | EsigCSP4-F | GTAGCGGCCACATGAAAAGT |
| EsigGOBP1-R | TCCAGCAATCCTCTTCTCGT |  | EsigCSP4-R | TCAAATGTACCTGGCGTTGT |
| EsigGOBP2-F | AGACGACGAGGCTTTCAAGA |  | EsigCSP5-F | TGCGAGACAACTTGTTGGAG |
| EsigGOBP2-R | TCTATATCCGTGGGCGTAGC |  | EsigCSP5-R | TTGATCTCCGGGTCATACTTG |
| EsigGOBP3-F | CTTGGACACCACCATCATTG |  | EsigCSP6-F | ACGATGATGTTGACGTGGAA |
| EsigGOBP3-R | GTCTTCTTCCGCAAAACTCG |  | EsigCSP6-R | ACTCGTCCTGCTGTGAACAA |
| EsigGOBP4-F | TGTATAACGCTCGTGGCATC |  | EsigOR1-F | GCGACAAGATCCGGTATGAA |
| EsigGOBP4-R | TGTCGTTTTCCGGTAACTCC |  | EsigOR1-R | ACTCTTCGCGTGAATGATCC |
| EsigGOBP5-F | TGCTTTAATGGCTGCTGTTG |  | EsigOR2-F | AGTCGTTGCCAGCTCATTCT |
| EsigGOBP5-R | TCCATACAGCCGACTTCCTT |  | EsigOR2-R | CCGACTCGCACTGTCTCATA |
| EsigGOBP6-F | CCACCAAGGACGCTAAATGT |  | EsigSNMP1-F | GACCCGTTGTCTGGAACAGT |
| EsigGOBP6-R | AGTCGAATCCGGTCACAATC |  | EsigSNMP1-R | CAGCAGTCCACAAGCGATAA |
| EsigOBP6-F | GAAGGGTGGCGATTACAAGA |  | EsigGR1-F | AGCGCGAACAAACAACTCTT |
| EsigOBP6-R | TCTCGTGGTAGCATTTGACG |  | EsigGR1-R | ACTTGGATTCGGGTTCCTCT |
| EsigOBP7-F | AGGGGCCAAGAAATAGTGGC |  | 18S-F | CAAGCACAACCGTCTCTTCA |
| EsigOBP7-R | TGTGTTGACCGCTTTCAGGT |  | 18S-R | ACCAGATCTTACGGGCCTCT |
| EsigOBP8-R | ACAAGACACGTCAGCACAGG |  |  |  |
| EsigOBP8-F | ATCGGCTCAACTCCACATTC |  |  |  |
