# Supplementary material for: Olfactory Proteins and Their Expression Profiles in the Eucalyptus Pest Endoclita signifier Larvae
Source: Front Physiol. 2021 Jul 19;12:682537. doi: 10.3389/fphys.2021.682537 (PMC8327093; doi:10.3389/fphys.2021.682537)
Supplement: Supplementary file 4 [file Table_4.DOCX]

Olfactory proteins and their expression profiles in the Eucalyptus pest *Endoclita signifie*r larvae

Xiaoyu Zhang^2^, Xiuhao Yang^3^, Hongxuan Ma^4^, Xiumei Liu^4^, Zhende Yang^1^, Ping Hu^1, 2*^

**Supplementary file 4 Best blastx hits for ionotropic receptors(IRs) of *Endoclita signifier***

| **Name** | **Nr description** | **Species** | **Acc. NO.** | **Cuticula FPKM** | **Head FPKM** | **Cuticula VS Head** |
| --- | --- | --- | --- | --- | --- | --- |
| EsigIR1 | ionotropic receptor IR14 | *Lobesia botrana* | AXF48845.1 | 1.7 | 0 | up |
| EsigIR75p-1 | putative ionotropic receptor IR75p.1 | *Hedya nubiferana* | AST36233.1 | 1.99 | 0 | up |
| EsigIR25a | putative ionotropic receptor IR25a | *Hedya nubiferana* | AST36228.1 | 0.2 | 1.26 | down |
| EsigIR2 | ionotropic receptor, partial | *Dioryctria abietella* | AZT78940.1 | 0.29 | 1.79 | down |
| EsigIR3 | ionotropic receptor IR33 | *Lobesia botrana* | AXF48864.1 | 0.11 | 1.18 | down |
| EsigIR40a-1 | ionotropic receptor 40a | *Hyposmocoma kahamanoa* | XP_026328485.1 | 1.34 | 0.63 | up |
| EsigIR75p-2 | putative ionotropic receptor IR75p.1 | *Hedya nubiferana* | AST36233.1 | 1.31 | 7.99 | down |
| EsigIR4 | ionotropic receptor IR13 | *Lobesia botrana* | AXF48844.1 | 7.74 | 14.91 | down |
| EsigIR76b | ionotropic receptor 76b | *Heortia vitessoides* | AZB49414.1 | 0 | 0 | no change |
| EsigIR93a-1 | ionotropic receptor 93a | *Bicyclus anynana* | XP_023954499.1 | 1.34 | 0 | up |
| EsigIR5 | glutamate receptor ionotropic, kainate 2-like | *Plutella xylostella* | XP_011566019.1 | 4.03 | 2.74 | up |
| EsigIR6 | glutamate receptor ionotropic, kainate 2-like | *Galleria mellonella* | XP_026749114.1 | 0.87 | 1.69 | down |
| EsigIR7 | glutamate receptor ionotropic, NMDA 2C isoform X2 | *Hyposmocoma kahamanoa* | XP_026316685.1 | 6.39 | 9.32 | down |
| EsigIR8 | glutamate receptor ionotropic, kainate 2 isoform X1 | *Aedes aegypti* | XP_021697648.1 | 2.59 | 4 | down |
